# Decoding functional hematopoietic progenitor cells in the adult human lung

**DOI:** 10.1182/blood.2024027884

**Published:** 2025-05-01

**Authors:** Catharina Conrad, Mélia Magnen, Jessica Tsui, Harrison Wismer, Mohammad Naser, Urmila Venkataramani, Bushra Samad, Simon J. Cleary, Longhui Qiu, Jennifer J. Tian, Marco De Giovanni, Nicole Mende, Andrew D. Leavitt, Emmanuelle Passegué, Elisa Laurenti, Alexis J. Combes, Mark R. Looney

**Affiliations:** 1 Department of Medicine, University of California, San Francisco; San Francisco, CA, USA; 2 UCSF CoLabs, University of California, San Francisco, CA USA; 3 Department of Microbiology & Immunology, University of California, San Francisco; San Francisco, CA, USA; 4 Wellcome – MRC Cambridge Stem Cell Institute, Department of Hematology, University of Cambridge; Cambridge, UK; 5 Columbia Stem Cell Initiative, Columbia University Irving Medical Center; New York, NY, USA; 6 Department of Pathology, University of California, San Francisco; San Francisco, CA, USA; 7 Bakar ImmunoX Initiative, University of California, San Francisco; San Francisco, CA, USA; 8 Biomedical Sciences Program, University of California, San Francisco, CA, USA; 9 Department of Laboratory Medicine, University of California, San Francisco, CA, USA

**Keywords:** Stem cell biology, Hematopoeitic stem and progenitor cells, Extramedullary hematopoeisis, Erythropoeisis, Stem cell transplantation, Blood and immune cell production

## Abstract

While the bone marrow is the main site of blood cell production in adults, rare pools of hematopoietic stem and progenitor cells have been found in extramedullary organs. In mice, we have previously shown that the lung contains hematopoietic progenitor cells and is a site of platelet production. Here, in the adult human lung, we show that functional hematopoietic precursors reside in the extravascular spaces with a frequency similar to the bone marrow, and are capable of proliferation and engraftment in mice. The gene signature of pulmonary and medullary CD34^+^ hematopoietic progenitors indicates greater baseline activation of immune, megakaryocyte/platelet and erythroid-related pathways in lung progenitors. Spatial transcriptomics mapped blood progenitors in the lung to an alveolar interstitium niche with only a few cells identified in an intravascular location. In human blood samples collected for stem cell transplantation, CD34^+^ cells with a lung signature enriched the mobilized pool of hematopoietic stem cells. These results identify the lung as a pool for uniquely programmed blood stem and progenitor cells with the potential to support hematopoiesis in humans.

## Introduction

Hematopoietic stem cells (HSCs) are self-renewing cells residing in the bone marrow (BM) that are ultimately responsible for production of all mature circulating blood cell lineages.^1^ Despite the burden of maintaining hematopoiesis, HSCs are a rare cell type in the BM accounting for <0.01% of nucleated cells.^2,3^ They occupy and are maintained by a specific BM niche yet are able to exit the BM environment and enter the circulation, although the explanation of this behavior is largely unknown.^4^ HSCs are central to the pathogenesis of serious disorders including myelodysplasia, acute and chronic leukemia, aplastic anemia^5^ and clonal hematopoiesis^6^, and HSC transplantation can be a life-saving therapy. Previously, we identified a population of hematopoietic stem and progenitor cells (HSPCs) in the mouse lung, but their function was unclear.^7^ Motivated by this background, in this study we sought to determine if HSPCs occupied the adult human lung—an organ with a vast vasculature that contains a wide-ranging repertoire of stromal and immune cells—and to determine the niche and function of these cells.

## Methods

### Human samples

Fresh human tissues were obtained from deceased organ donors after either brain death (DBD) or circulatory death (DCD) (Table S1). All patients were on mechanical ventilation and treated in the intensive care unit until organ retrieval. Lungs and vertebral bodies were surgically recovered and immediately placed on ice. Peripheral blood (PB) was collected in sodium heparin tubes. Healthy human stem cell donors were treated with a G-CSF mobilization regimen and peripheral blood stem cells were collected by apheresis. We received 1 mL of the mononuclear fraction that would have otherwise been discarded. Isolation of cells, magnetic enrichment, antibody staining, cell sorting and immune phenotyping by flow cytometry are described in the Supplemental Methods.

### Cell culture assays

Lineage depleted BM or lung cells were plated in MethoCult™ or MegaCult™-C medium according to the manufacturer’s instructions. After incubation for 10-14 days at 37°C in 5% CO_2_, hematopoietic colonies were scored based on morphological and phenotypic criteria and quantified by manual counting using brightfield microscopy. Further details are described in the Supplemental Methods.

### Xenotransplantation

NSG-SGM3 mice (NOD.Cg-Prkdc^scid^ Il2rg^tm1Wjl^ Tg(CMV-IL3, CSF2, KITLG)1Eav/MloySzJ, Stock No: 013062) were purchased at the Jackson Laboratory and housed in a Specific-Pathogen-Free (SPF) animal facility. One day before reconstitution, mice were pre-conditioned by sublethal irradiation (2.4 Gy).^8^ On the day of transplantation, Lineage negative (Lin^-^) lung and BM cells were thawed and 1.5 x10^6^ viable cells were intravenously injected into the tail vein of recipient mice.^8^ Recipient mice were transplanted with matched lung and BM cells where possible. 10 weeks post-transplantation, the BM, lung, and blood were investigated for engraftment of human cells. Futher details are described in the Supplemental Methods.

### scRNA-seq

Live, Lin^-^ CD34^+^ cells from the lung and BM were sorted for subsequent 10x Genomics scRNAseq. Live, Lin^+^ cells were collected for demultiplexing the samples from different individuals based on single nucleotide polymorphisms (SNPs).^9^ Further details of scRNA-seq workflow and the bioinformatic analysis are provided in the Supplemental Methods.

### Spatial transcriptomics

Human lung tissue from deceased organ donor samples was frozen in OCT on dry ice and tissue blocks were processed at the facility of Resolve Biosciences, San Jose for spatial transcriptomics using a custom 100 gene panel. Futher details on spatial transcriptomics workflow and the bioinformatic analysis are provided in the Supplemental Methods.

### Statistical analysis

Statistical analysis was done with GraphPad Prism version 10.0.2, R/RStudio version 4.0.3, or Python version 3.12.1. Statistics for each analysis are describe in the relevant section.

## Results

### The adult human lung contains functional HSPCs

**Figure 1:**
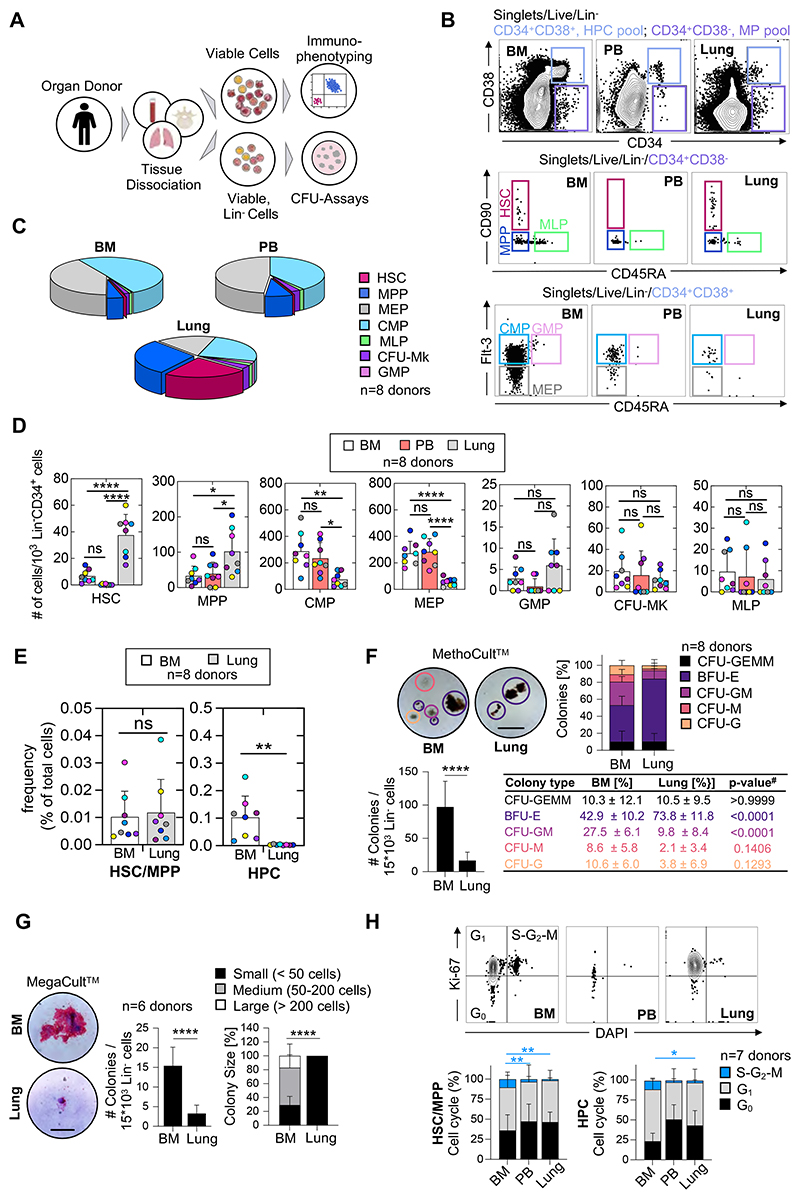
The human lung contains phenotypic hematopoietic progenitors with *in vitro* proliferation and differentiation capacity. **(A)** Pipeline for flow-cytometric immunophenotyping and evaluation of *in vitro* colony-forming capacity of hematopoietic progenitor cells from BM, lung and PB of organ donors. **(B)** Normalized flow cytometry plots of BM, PB in the Live/Lin^-^ gate from a representative donor showing stem cell subsets within the multipotent (MP [CD34^+^CD38^-^], light purple) and the hematopoietic progenitor cell (HPC [CD34^+^CD38^+^], light blue) pool. HSC, hematopoietic stem cell; MPP, multipotent progenitor; MLP, multilymphoid progenitor; CMP, common myeloid progenitor; MEP, megakaryocyte-erythroid progenitor; GMP, granulocyte-macrophage progenitor; CFU-Mk, colony-forming-unit megakaryocyte. **(C)** Composition of hematopoietic progenitor subsets in the BM, PB and lung (n=8). **(D)** Numbers of hematopoietic progenitor cell subsets in the BM (white), PB (red) and lung (grey) per 10^3^ Lin^-^CD34^+^ cells. N=8 donors, bars indicate mean number of cells ± SD, colors of the dots represent individual donors. ANOVA followed by Sidak’s multiple comparison test, *p<0.03; **p<0.002; ***p<0.0002; ****p<0.0001; ns, not significant. **(E)** Frequency of HSCs/MPPs and HPCs as a percentage of total nucleated cells in the lung or BM, respectively. Dot colors represent individual donors, bars indicate mean ± SD. Student’s t-test, **p<0.01; ns, not significant. **(F)** Culture initiating capacity of lung and BM progenitors in MethoCult™ (n=8): Representative colonies (scale bar, 500μm), colony composition and colony quantity for progenitors derived from the BM and lung. Student’s t-test, ****p<0.0001, ^#^ANOVA followed by Sidak’s multiple comparison test. CFU, colony-forming unit; BFU-E (purple), burst-forming unit-erythroid; G (orange), granulocyte; M (red), macrophage; GM (pink), granulocyte macrophage; GEMM (black), granulocyte, erythroid, macrophage, megakaryocyte. **(G)** Culture initiating capacity of lung and BM progenitors in MegaCult™ (n=6): Representative colonies (scale bar, 100μm), colony quantity and colony size for progenitors from the BM and lung. Bar graph represents mean number of colonies ± SD, Student’s t-test, ****p<0.0001. Stacked bars represent mean proportion ± SD, Kruskal-Wallis test, ****p<0.0001. **(H)** Proportions of cycling (S-G2-M phase (blue), Ki-67^+^DAPI^+^), preparing/growing (G1 (grey), Ki-67+DAPI^-^) and resting cells (G0 (black), Ki-67^-^DAPI^-^) in the HSC/MPP and HPC pool from BM, PB and lung (n=7). Stacked bars represent mean proportion ± SD, ANOVA followed by Sidak’s multiple comparison test, **p<0.01, *p<0.05. For comparisons not indicated, no statistically significant differences were observed.

We had the unique opportunity to receive matched adult human lungs, vertebral bodies (BM), and peripheral blood (PB) freshly recovered from deceased research donors (Table S1). The lungs were extensively perfused at the time of collection, and we selected healthy-appearing lung tissue for experiments (Supplemental Figure 1A). After tissue dissociation and the rendering of single-cell suspensions, we characterized live, lineage negative (Lin^-^) cells (Figure 1A) using standard surface markers for HSPCs (Table S2).^10–12^ Notably, our lineage panel contained markers for mature immune, endothelial, and epithelial cells, allowing for the phenotyping of rare Lin^-^CD34^+^ cells. We discovered a distinct population of multipotent progenitors (MP; (CD34^+^/CD38^-^) in the lung and BM and very few of these cells in the peripheral blood (PB) (Figure 1B). The lung and BM contained cells with surface staining consistent with HSCs (CD34^+^/CD38^-^/CD90^+^/CD45A^-^) but these cells were absent in the PB. Multipotent progenitor (MPP) cells (CD34^+^/CD38^-^/CD90^-^/CD45A^-^) were observed in all three tissues. CMP (CD34^+^/CD38^+^/CD45A^-^/Flt-3^+^), GMP (CD34^+^/CD38^+^/CD45A^+^/Flt-3^+^), and MEP (CD34^+^/CD38^+^/CD45A^-^/Flt-3^-^) cells were observed in all three tissues but were less common in the lung (Figure 1B-C). Overall, the BM and PB had nearly identical proportions of hematopoietic progenitors, while Lin^-^ lung cells were enriched for immunophenotypic HSCs and MPPs (Figure 1C-D).

To rule out residual blood as a source of HSPCs in perfused lungs, we estimated the numbers of progenitors in equivalent volumes of blood and lung tissue, yielding lung HSPC numbers that could not be explained by retention of intravascular blood in lungs (Supplemental Figure 1B). Further, the distinct proportions of cell subsets in lung and blood indicates a tissue-specific progenitor composition. Remarkably, the frequency of the immunophenotypic multipotent progenitors in the lung is similar to the BM (Figure 1E), while the pool of more committed hematopoietic progenitor cells is much smaller in the lung (Figure 1E). We also tested for the effects of donor age and gender on our results and found that increasing age was associated with fewer numbers of HSPCs in the BM but not in the lung (Supplemental Figure 1C). Gender was not associated with changes in HSPC frequency in the BM or lung (Supplemental Figure 1D).

Since fibroblasts are generally lineage-negative cells, but some can be CD34-positive^13^, we included a marker for fibroblasts (PDGFRα) in our lineage panel to determine if fibroblast contamination could affect our results, but we did not detect any changes in HSPC frequencies between our two lineage panels (Supplemental Figure 2A-C).

We next tested the functional capacity of lung HSPCs using *in vitro* colony forming assays. We plated Lin^-^ cells from the lung or BM in MethoCult™ and observed a variety of colonies from both tissues. The Lin^-^ cells from the lung produced overall fewer colonies, but with a significant increase in the relative proportion of erythroid colonies (BFU-E) (Figure 1F, Supplemental Figure 3A). Following morphologic colony assessment, we used flow cytometry (Supplemental Figure 3B-D) to confirm cellular colony composition and found that lung colonies were enriched with cells expressing the erythrocyte marker GlyA (Supplemental Figure 3D). There were not enough Lin^-^ cells from the PB to perform a comparative study, so we determined the CFU potential of nucleated blood cells. This yielded few colonies (Supplemental Figure 3E-G), including only a few BFU-E colonies, which is the predominant colony type produced by the lung. These results strengthen our conclusion that the presence of lung HSCs is not an artifact of blood contamination. We also plated Lin^-^ cells in MegaCult™ to test the potential to produce megakaryocytes, a cell population that we previously described as resident cells in the mouse lung.^7^ Both BM and lung cells were capable of producing megakaryocyte colonies although the lung produced fewer and small colonies (Figure 1F). Additionally, we sorted lung and BM HSCs and HPCs into MegaCult™ and observed that lung HSCs produced comparatively more megakaryocytes (Supplemental Figure 4A), while in BM, most cells with MK-CFU capacity are derived from precusors in the HPCs (Supplemental Figure 4B).

Overall, the fewer colonies observed from lung cells may also relate to reduced cell cycling compared to the BM (Figure 1H). The molecular cues that regulate the transition to activity in pulmonary HSPCs are not known and could be different from growth factors added in classical *in vitro* CFU assays. Together, we conclude that the human lung contains functional HSPCs that exhibit a bias towards erythroid and megakaryocyte lineages within the hematopoeitic progenitor tree.

### Human lung-derived hematopoietic progenitors have engraftment potential

**Figure 2:**
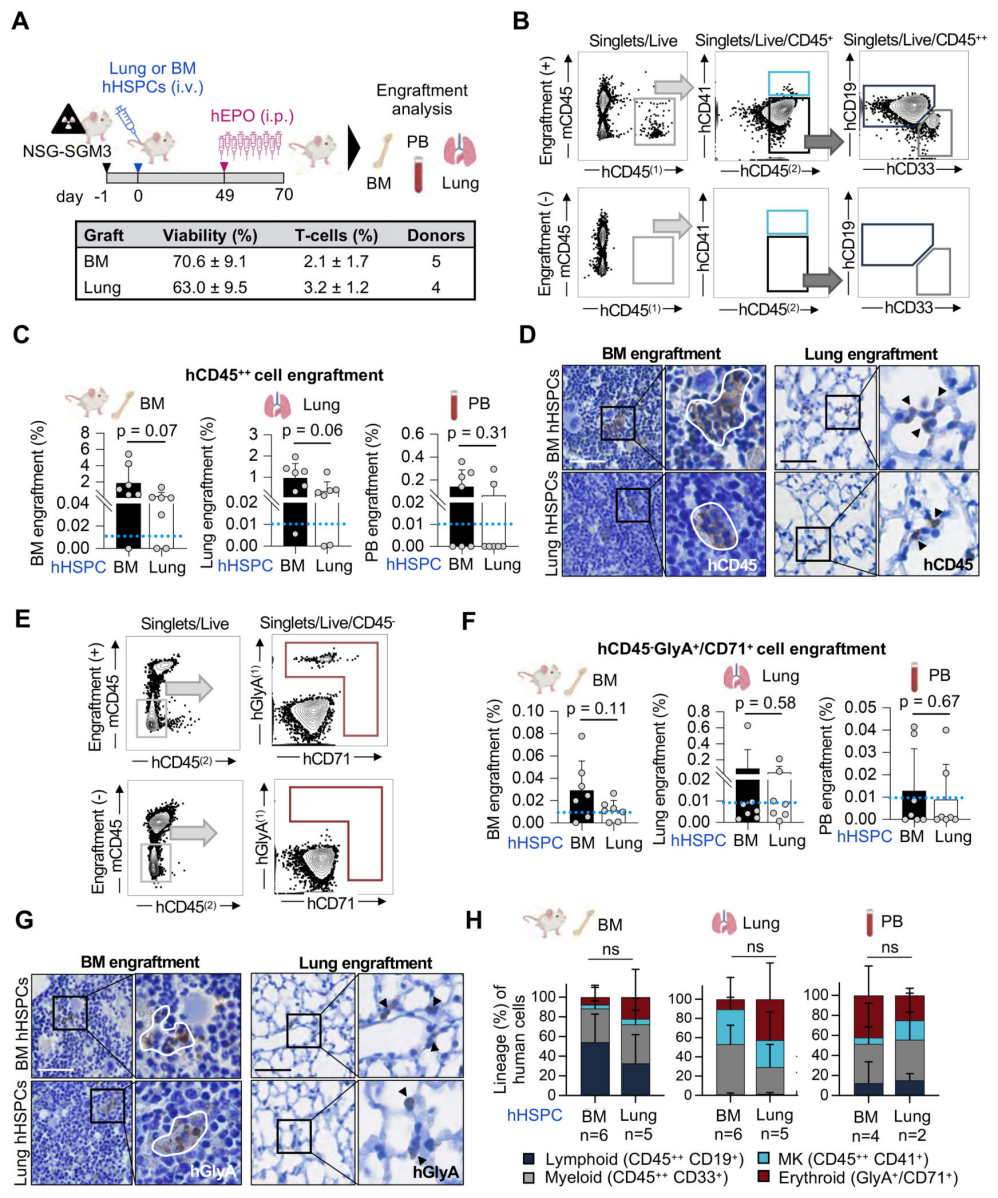
Human lung-derived hematopoietic progenitors have *in vivo* engraftment potential. **(A)** Experimental procedure to compare the *in vivo* engraftment efficiency of lung and BM hematopoietic progenitors: After sublethal irradiation, NSG-SGM3 mice were injected i.v. with either 1.5 x10^6^ Live/Lin^-^ human lung or BM cells. Ten weeks post transplantation, the BM, PB and lung of recipient mice were collected and investigated for human cell engraftment. The graft properties are summarized in the table below. **(B)** Representative flow plots of human myeloid (hCD45^++^, hCD33^+^) and lymphoid (hCD45^++^, hCD19^+^) cell engraftment in the BM of a recipient mouse (+, upper panel) and non-transplanted control (-, lower panel). **(C)** Engraftment efficiency of human cells after xenotransplantation measured by flow cytometry. Bar graphs represent the percentage of hCD45^++^ cell engraftment in the BM, lung, and PB of recipient mice after transplantation of HSPCs from human BM (black) or lung (white). Mean ± SD; Student’s t-test values are given, individual data points for each animal are plotted as gray dots. Blue dotted line indicates threshold for positive engraftment. **(D)** Detection of human cells in the BM (left panel) and lung (right panel) of recipient mice by immunostaining against human CD45 (hCD45). Scale bar, 50μm. **(E)** Representative flow cytometry plots of human erythroid (CD45^-^, hGlyA^+^ or hCD71^+^) cell engraftment in the BM of a recipient mouse (+, upper panel) and non-transplanted control (-, lower panel). **(F)** Human erythroid cell expansion (CD45^-^, hGlyA^+^ or hCD71^+^) in the BM, lung and PB of recipient mice measured by flow cytometry. Bar graphs representing the percentage of human CD45^-^GlyA^+^CD71^+^ cells in BM, lung, and PB after transplantation of HSPCs from human BM (black) or lung (white). Mean ± SD; Student’s t-test values are given, individual data points for each animal are plotted as gray dots. Blue dotted line indicates threshold for positive engraftment. **(G)** Detection of human erythroid cells in the BM (left panel) and lung (right panel) of recipient mice by immunostaining against human GlyA (hGlyA). Scale bar, 50μm. **(H)** Proportion of lineage expansion across all human cells detected in the BM, lung and PB, respectively. Stacked bars represent mean proportion ± SD, ANOVA followed by Sidak’s multiple comparison test, ns, not significant.

We next tested whether HSPCs isolated from the lung are capable of engraftment when xenotransplanted into immunodeficient mice.^8^ We chose NSG-SGM3 mice (human SCF, GM-CSF, IL-3) to support human myeloid cell engraftment^14^ for these experiments in which we adoptively transferred magnetic-enriched Lin^-^ cells from the lung or BM into mice after sublethal irradiation.^15^ Given that the production of erythroid cells in xenograft models is not reliable due to the lack of cross-reactivity between mouse EPO and the human EPO-receptor, mice received recombinant human erythropoietin injections in the final three weeks of the experiment (Figure 2A) as described previously.^8^ Human cells in the BM, lung and PB of recipient mice were assessed after 10 weeks, a time point that is commonly used to measure human HSC activity *in vivo*.^15–17^ Engraftment was rigorously defined as the presence of human CD45^+^ cells, using two different antibody clones, with the threshold for engraftment set to ≥0.01% CD45^++^ cells of all CD45^+^ cells (mouse and human) with at least 30 cells recorded in the CD45^++^ gate for BM and lung, and ≥ 15 cells for PB^8^ given that low levels of engrafted human cells are expected from previous studies.^17^ Examples of positive and negative engraftment (sublethal irradiation without adoptive cell transfer) are shown in Figure 2B. Overall, 6/7 mice with BM-derived HSPCs and 5/7 mice with lung-derived HSPCs engrafted in the BM and in the lung tissue (Figure 2C, Supplemental Figure 5A). In the PB, engraftment was observed in 4/7 mice with BM-derived HSPCs and 2/7 mice with lung-derived HSPCs (Figure 2C, Supplemental Figure 5A). Examples of BM- or lung-derived cells in the mouse BM or lung are shown in Figure 2D.

We also determined erythroid engraftment using the gating strategy in Figure 2E to detect CD45^-^, hGlyA^+^, hCD71^+^ cells and observed similar erythroid engraftment of BM- or lung-derived HSPCs in the mouse BM, lung, or PB (Figure 2F-G, Supplemental Figure 5B). Similar to BM-derived HSPCs, lung-derived HSPCs were capable of multilineage blood cell production (Figure 2H, Supplemental Figure 5C-E).

### Human lung HSPCs have unique transcriptional programming

**Figure 3:**
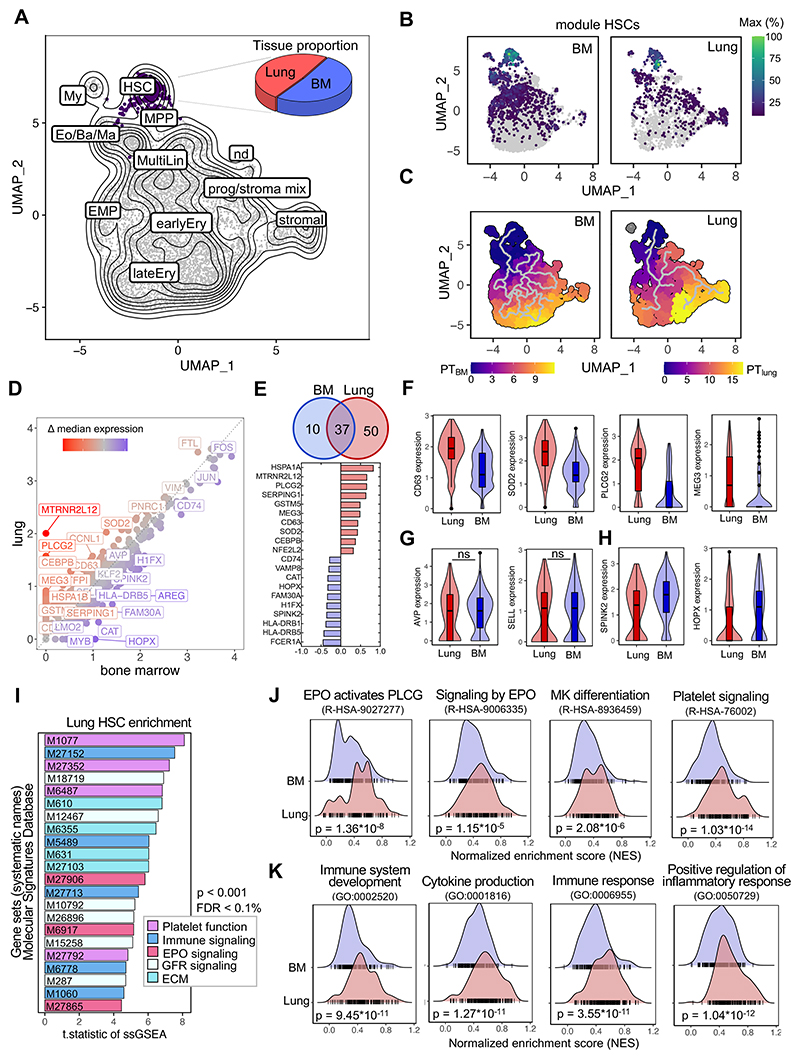
Comparative transcriptomic analysis of lung and BM HSCs reveals shared and unique gene expression profiles. **(A)** UMAP projection of BM and lung Lin^-^CD34^+^ progenitor hierarchy from 8 human donors highlighting the HSC/MPP cluster (purple). The pie graph indicates the proportion of cells from the BM (blue) and lung (red) within the multipotent progenitor subset. HSC/MPP, hematopoietic stem cell/multipotent progenitor; My, myeloid cell; Eo/Ba/Ma, eosinophil/basophil/mast cell progenitor; MultiLin, multi-lineage; EMP, erythroid megakaryocytic progenitor; earlyEry, early erythroid progenitor; lateEry, late erythroid progenitor; prog/stroma mix, progenitor stroma cell mix; nd, not determined. **(B)** Grouping of gene expression patterns into modules using Monocle3. Aggregate expression values of genes in the module highly specific for HSCs (Supplemental Figure 7) are shown individually for the BM and lung. **(C)** Pseudotime calculation for each cell within the BM and lung using Monocle3 to infer progression through different cellular differentiation to provide insights into the developmental trajectory. **(D)** Scatter plot of median gene expression of cells in the HSC/MPP cluster from the lung (red) and BM (blue) to visualize consistent (grey) and differentially (highlighted) expressed genes. **(E)** Venn diagram and top 10 differentially expressed genes. The number in each circle represents the amount of differentially expressed genes between lung (red) and BM (blue), the overlapping number indicates mutual differentially expressed genes based on the Wilcoxon ran-sum test in Seurat’s ‘FindMarkers’ function. **(F)** Box and violin plots showing the distribution of selected genes upregulated in pulmonary hematopoietic progenitor cells. Wilcoxon adjusted p-value <0.001. **(G)** Selection of marker genes shared between lung and BM as box and violin plots, respectively. ns, not significant. **(H)** Box and violin plots showing the distribution of markers genes upregulated in BM HSCs, Wilcoxon adjusted p-value <0.001. **(I)** T.statistic of ssGSEA scores for selected gene sets (Hallmark, Reactome, Biocarta, KEGG) enriched in pulmonary HSCs categorized by recurring functions. EPO, erythropoietin; ECM, extracellular matrix, FDR, false discovery rate; GFR, growth factor receptor; ssGSEA, single-sample Gene Set Enrichment Analysis. **(J)** Enrichment ridge plots comparing the distribution of enrichment scores in HSCs from lung (red) and BM (blue) of selected Reactome pathways. Rug plots indicate the scores of individual cells along the ridge plot. P-values are given in the figure, FDR R-HSA-9027277 = 2.38*10^-4^; FDR R-HSA-9006335 = 0.09; FDR R-HSA-8936459 = 0.03; R-HSA-76002 = 2.03*10^-10^. **(K)** Enrichment ridge plots showing the distribution of enrichment scores in lung (red) and BM (blue) with individual cell placement on the rug plot to compare selected GOBP (Gene Ontology Biological Process) gene set enrichments. P-values are given in the figure, FDR GO:00025 = 1.77*10^-6^; FDR GO:0001816 = 2.42*10^-7^; FDR GO:0006955 = 6.70*10^-7^; GO:0050729 = 2.02*10^-8^.

The availability of matched lung and medullary HSPCs allowed us to directly compare their transcriptional profiles using single-cell RNA-sequencing (scRNAseq, Supplemental Figure 6A). Our gating strategy is shown in Supplemental Figure 6B-C. We integrated all samples using Harmony^18^ and generated a batch-corrected UMAP to identify clusters of transcriptionally similar cells. We filtered out stromal and mesothelial cell populations and subsetted progenitor cells (Supplemental Figure 6C-E). Using the function ‘findConservedMarkers’, we identified genes that were consistently expressed across BM and lung-derived cells and annotated clusters based on a reference data set.^19,20^ Dimensionality reduction yielded a visual representation consistent with HSC and MPP production of progenies with progressive commitment to more differentiated fates (Figure 3A, Supplemental Figure 6F-G). To validate our annotation, we generated co-regulated modules of differentially expressed genes using Monocle3.^21^ The module highly specific for HSCs contains genes such as *AVP, SPINK2, SELL* and *HOPX* and was strongly expressed in cells from both the BM and lung (Figure 3B, Supplemental Figure 7A-B). We ordered the cells along a pseudotime trajectory to reconstruct their developmental path for each tissue individually, suggesting a relationship between HSCs and stromal cells in the lung that was absent in cells derived from the BM (Figure 3C). As previously discussed, some pulmonary fibroblasts are CD34^+^ and these results could point to the ontogeny of a subset of lung stromal cells.

Next, we compared the differential gene expression between medullary and lung cells within the HSC/MPP cluster and plotted the median expression of both cells on a scatter plot (Figure 3D). Using the Wilcoxon rank-sum test in Seurat’s ‘FindMarkers’ we identified 50 genes that were upregulated in lung HSCs and 10 genes that were higher in BM cells (Figure 3D). Among the top upregulated genes in lung HSCs, several genes (*CEBPB, SOD2, PLCG2, HSPA1A*) were associated with maintaining hematopoietic stem cell quiescence and fitness (Figure 3E).^22–25^ We attributed the highest biological relevance to genes that were upregulated in most of the cells, as validated by analyzing the distribution of gene expression values (Figure 3F-H). HSCs from the lung have unique features (Figure 3F) and share characteristics of the hematopoietic lineage (Figure 3G), while as expected, cells from the BM have high expression of classical stem cell genes (Figure 3H).^19^

Next, we performed single-sample Gene Set Enrichment Analysis (ssGSEA) to identify pathways that are differentially regulated between cells from the lung and the BM. The enrichment scores were calculated across all individual cells and tissues for gene set collections from the Molecular Signature Database (H: hallmark, CP: canonical pathways, C5: ontology). In the gene sets analyzed, repeatedly pathways associated with erythropoietin (EPO) signaling, platelet function, and immune responses were found to be upregulated in pulmonary HSCs (Figure 3I). Side-by-side comparison of selected pathways indicates that HSCs from the lung are enriched for megakaryocyte (R-HSA-8936459) and EPO-induced erythroblast (R-HSA-9027277, R-HSA-9006335) differentiation, as indicated by higher normalized enrichment scores (NES) (Figure 3J). Our finding of increased EPO signaling pathways in lung HSCs is consistent with our data in Figure 1E on the erythroid-biasing of lung colonies. Our finding of platelet and megakaryocyte-skewing of lung HSCs is provocative in light of previous work on platelet production in the lung and tissue-resident immune-like megakaryocytes.^7,26,27^ Additionally, we found inflammatory signaling to be upregulated in pulmonary HSCs (Figure 3K), suggesting that these cells could impart unique immunological functions of their progeny.

A small cluster of cells has recently been suggested as hematopoetic stem cell population in an analysis of the healthy and diseased human lung tissue based on *CD34, SPINK2, STMN* and *PRSS57* expression^28^, but the self-renewal and differentiation potential, and the location of these cells is not known. Also, CD34^+^ cells can be found in endothelial, lymphatic and fibroblast clusters (Supplemental Figure 8A-B). To identify potential HSCs based on their transcriptomic profile, we used UCell scoring to find Lin^-^, CD34^+^ cells with an HSC signature in a dataset combining 9 human lung scRNAseq studies (Human Lung Cell Atlas V2, HLCA^29^) (Supplemental Figure 8C-D). We projected these cells on our UMAP structure of hematopoietic stem and progenitors cells from the lung and BM (Figure 3A) in Supplemental Figure 8E and mapped 43 cells to the HSC/MPP cluster (Supplemental Figure 8F) across all integrated datasets in the HLCA (Supplemental Figure 8G). We conclude that given their rarity and the co-expression of CD34 in multiple lung cell types, lung HSCs are mostly masked and overlooked when using standard unsupervised clustering techniques.

### HSPCs in the lung reside in the extravascular tissue

**Figure 4:**
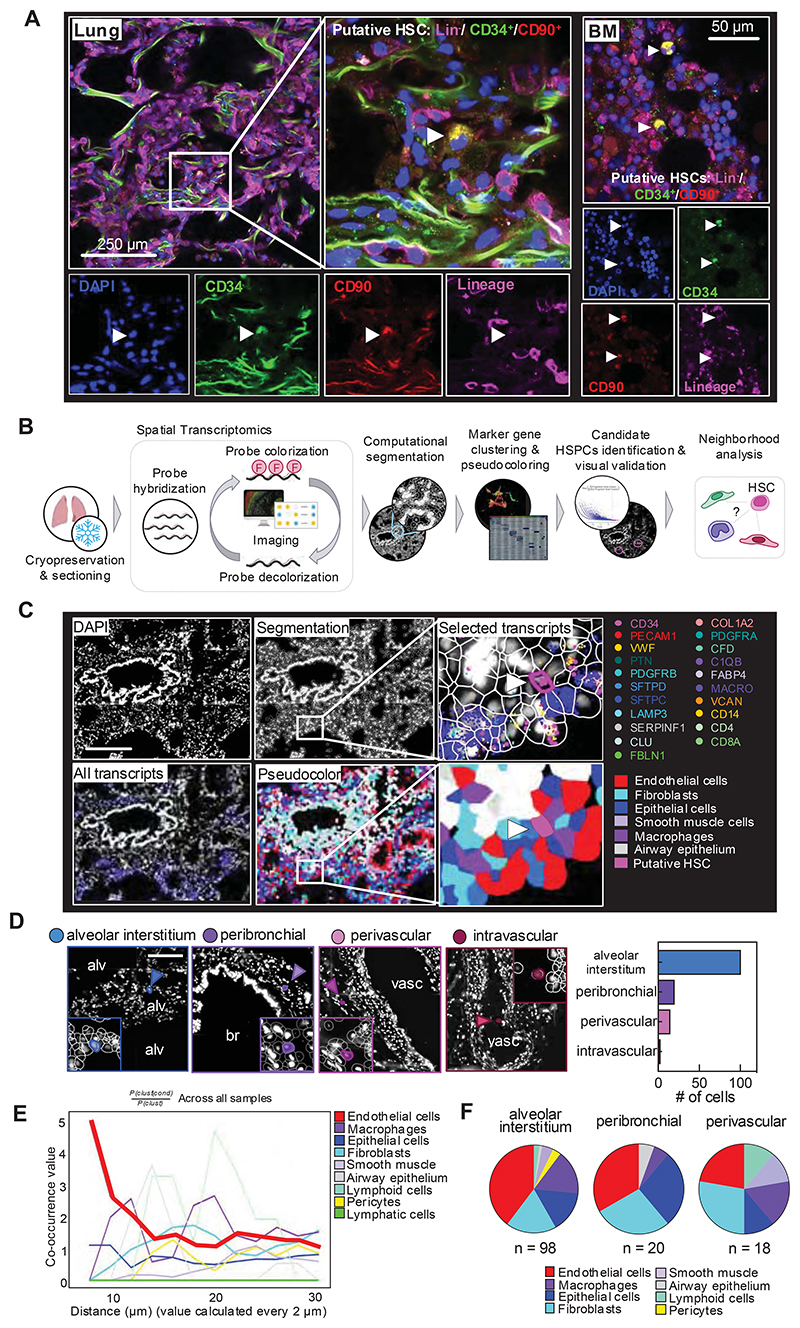
Spatial mapping of phenotypic CD34^+^ HSPCs in the lung. **(A)** Immunofluorescence imaging of putative HSCs (Lin^-^/CD34^+^/CD90^+^) in the human lung and BM. Left panel: Representative section of lung showing a Lin^-^/CD34^+^/CD90^+^ in the interstitial space. Right panel: Representative section of BM showing two Lin^-^/CD34^+^/CD90^+^ cells. **(B)** Spatial transcriptomics analysis workflow. smFISH was performed to visualize gene expression in human lung tissue. Transcripts were assigned to individual cells after cell segmentation and cells were annotated based on marker gene expression (Supplemental Figure 10A-D). HSPC candidate cells were computationally identified based on their gene signature and visually validated (Supplemental Figure 10E-F, Supplemental Figure 11A-B). **(C)** Representative image of a putative HSPC in its pulmonary niche. Upper panel (left to right): DAPI staining, QuPath segmentation, zoom on putative HSC (arrow). Selected transcripts are shown. Lower panel (left to right): all transcripts, pseudo-coloring of cell types in the lung tissue based on marker clustering (Supplemental Figure 10). Zoom on putative HSPC in niche. Scale bar, 250 μm. **(D)** Anatomic location of candidate cells in the lung. Representative images of phenotypic HSPCs in four major locations (alveolar interstitium, peribronchial, perivascular or intravascular) and proportion of cells in each location. Alv, alveolar space; br, bronchus; vasc, vasculature. Scale bar, 150μm. **(E)** Squidpy co-occurrence score computed every 2 μm between putative HSPCs and the rest of the clusters across lung tissue sections from 4 organ donors. High score values indicate greater co-occurrence probability; endothelial cells (red) co-occur with the HSPCs at short distances. **(F)** Pie graphs showing the proportion of neighboring cells within a radius of 20 μm from the putative HSPCs in the major anatomic locations.

The lung is composed of diverse cell entities, such as epithelial, endothelial, stromal, and immune cell subpopulations that could provide a unique niche for HSPC maintenance and differentiation.^20^ We used immunostaining to localize putative HSCs (Lin^-^/CD34^+^/CD90^+^) in human lung and BM and found HSCs that localized to the alveolar interstitium (Figure 4A, Supplemental Figure 9A-B). To characterize the pulmonary HSC niche, we used a spatial transcriptomics approach based on combinatorial single-molecule fluorescent *in situ* hybridization (Figure 4B). We designed a marker panel that characterizes HSPCs as well as the common cell entities of the lung (Table S3). Following QuPath-based cell segmentation, we annotated the lung cells by performing unsupervised clustering on transcript expression values (Figure 4C, workflow shown in Supplemental Figure 10A-D). We filtered for putative HSPCs defined by CD34^+^ positivity, expression of HSPC-associated transcripts and negativity for marker genes of other lung cell entities (Figure 4C, workflow shown in Supplemental Figure 10E-F, see also Supplemental Figure 11A-B). We visually validated all candidate cells and assigned them to their anatomic location. Over 90% of cells matching the criteria for HSPCs localized to the extravascular lung, with the majority of the cells in the alveolar interstitium or in proximity of bronchi (peribronchial) and vasculature (perivascular) (Figure 4D, Supplemental Figure 11A). To define the neighborhood of HSPCs on the cellular level, we used Squidpy for co-occurrence analysis across all samples^30^ suggesting that the immediate HSPC niche is mainly formed by endothelial cells, although epithelium and fibroblasts have a steady presence (Figure 4E-F, Supplemental Figure 11C).

### HSCs with pulmonary signatures are mobilized during apheresis collections for stem cell transplantation

**Figure 5:**
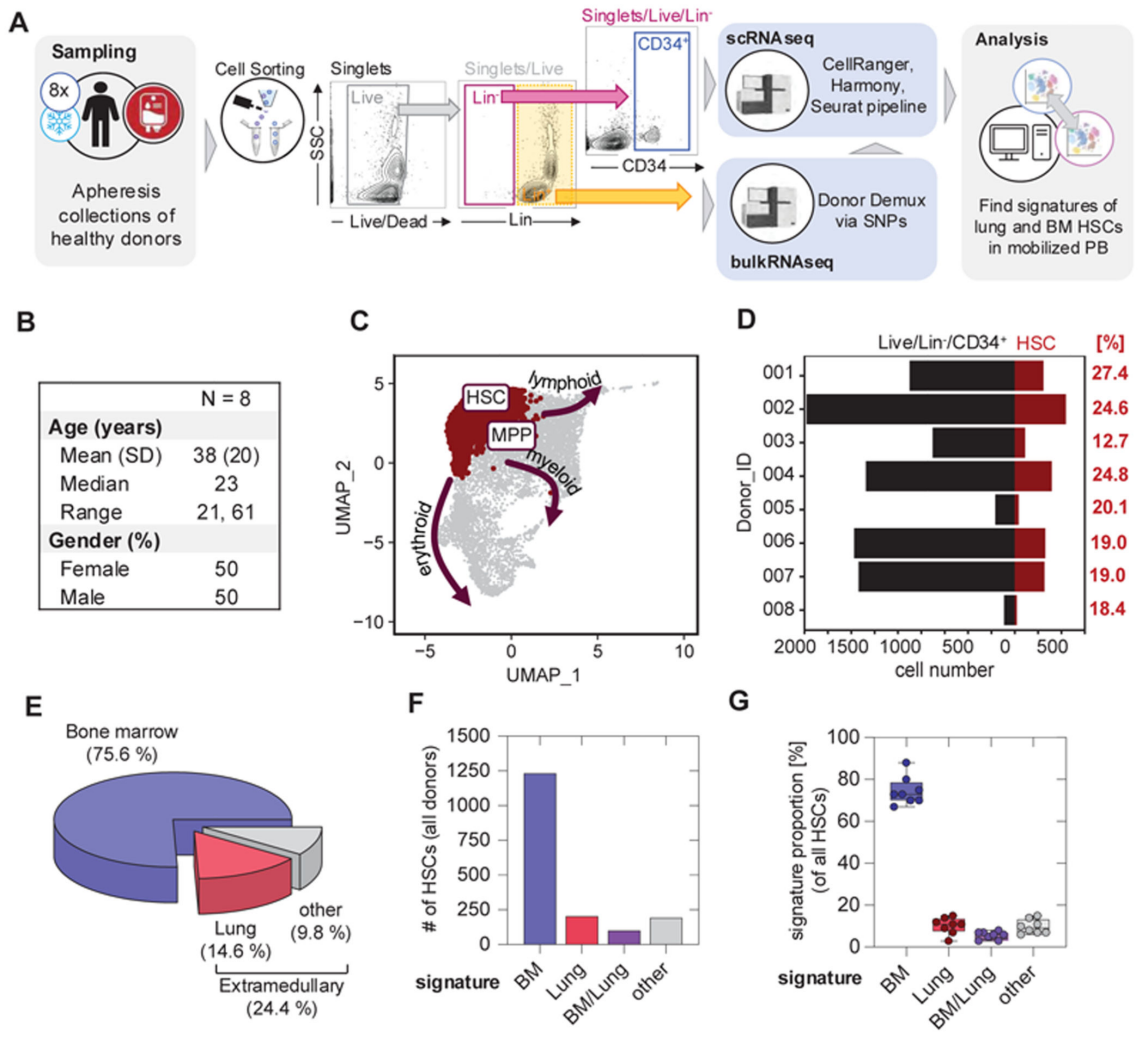
HSCs with pulmonary signatures are mobilized during apheresis collections for transplantation. **(A)** Peripheral blood stem cells of 8 healthy donors given G-CSF for mobilization were collected via apheresis and cryopreserved (Sampling). Live/Lin^-^/CD34^+^ cells were flow sorted and encapsulated, 10x Chromium™ Single 3’ v2 libraries were prepared, pooled and sequenced (scRNAseq). For donor demultiplexing via SNPs, bulkRNAseq was performed on Live/Lin^+^ cells (bulkRNAseq). Following Louvain clustering and annotation, phenotypic HSCs were subsetted from the mobilized pool and examined for their expression of canonical, lung and BM HSC signature genes using UCell. **(B)** Basic demographics of the donor population. **(C)** Batch corrected UMAP representation highlighting the HSC/MPP cluster (red), arrows indicate developmental trajectory into more committed progenies (erythroid, myeloid, lymphoid). **(D)** Total number of progenitor cells and number of HSCs per donor. Fraction of HSCs among all cells is given in red. **(E)** Pie graph showing the proportions of medullary (blue) and extramedullary (lung, red; other, grey) signatures in the HSC fraction of apheresis samples. **(F)** Bar graph showing the absolute numbers of HSCs across all donor that had a unique BM (blue) or lung (red) signature, cells that exhibited features of both BM and lung (violet) and cells that could not be assigned to either of these categories (grey). **(G)** Box and Whisker plot representing the percentage of medullary and extramedullary signatures identified the HSC population. Dots represent the individual allogeneic donors.

Finally, we evaluated the potential function of pulmonary HSCs by evaluating their contribution to the mobilized blood progenitor pool. In 8 healthy volunteers undergoing mobilized blood donations for stem cell transplantation (apheresis donations), we analyzed the transcriptomic profile of Live/Lin^-^/CD34^+^ cells by scRNAseq (Figure 5A-B). Following cell annotation (Figure 5C), we subsetted the HSCs per donor (Figure 5D) and calculated tissue-specific signature scores using UCell to infer their source (Table S4). We determined that approximately 25% of mobilized HSCs were of extramedullary origin including approximately 15% of total cells being of pulmonary origin (Figure 5E-G). These results indicate that extramedullary HSCs are mobilized into the HSC pool used for transplantation and points to their potential biological function.

## Discussion

For many years, HSCs were viewed as unbiased cells that initiated hematopoietic development and specialization. Enabled by new technologies, our understanding of hematopoiesis has been refined to include the possibility that developmental biases may be present even in these most undifferentiated cells, such as with megakaryocyte-biased HSCs.^11^ However, the mechanisms responsible for this early biasing or specialization are not clear.^31,32^ Here, we propose that the traditional view of HSC residency in the BM should be reconsidered to include extramedullary tissues, such as the lung. Indeed, and remarkably, we found an equal frequency of multipotent progenitors residing in the bone marrow and lung. Due to the low frequency, we used approaches to enrich for the presence of HSPCs in our studies, which were not done in previous studies and likely enabled the profiling of this rare subset of cells amongst the >30 different lung cell types.

It is clear from our studies that the lung HSPCs have unique features compared to their medullary counterparts, but also to HSPCs found at other extramedullary sites (peripheral blood and spleen^33^). Chief amongst these, and perhaps obvious from our understanding of lung biology, is that lung HSPCs, like spleen HSPCs, are less active in terms of cell division and the production of more differentiated and specialized hematopoietic cells than their BM counterparts.^33^ These results from *in vitro* experiments would suggest that lung HSPCs function as a reserve pool that could be mobilized in the setting of hematopoietic stress, or as we have shown here, during stem cell collections for transplantation. Further studies are needed to define physiological and pathological stimuli that trigger hematopoiesis in the lung.

Our xenotransplantation experiments indicated that lung HSPCs performed similarly to medullary HSPCs, when given the challenge of engraftment after sublethal irradiation. A wide range of engraftment levels and analysis time points have been used for measuring human HSC activity *in vivo* and there is no standard in the field.^17^ Thus, despite low graft expansion in our model (NSG-SGM3 mice, sub-lethal irradiation, intravenous HSPCs), our data are consistent with previous outcomes and demonstrate self-renewal capacity of pulmonary HSPCs *in vivo*. Additionally, we included donors of any age and sex (Table S1), which might have contributed to larger variations in our results.

Our transcriptomics analysis revealed a distinct molecular program in lung HSPCs that was further distinguishing compared to medullary HSPCs. We found clear megakaryocyte biasing of lung vs. medullary HSPCs indicating that perhaps the lung is a source of these biased progenitors. Our colony analysis of lung HSCs also supports megakaryocyte biasing. This is an intriguing finding given (1) the role of the lung in platelet biogenesis^7^ and (2) the presence of tissue resident immune-like megakaryocytes in the lung that may be derived from a lung progenitor.^26,27^ We further found an erythroid bias of lung HSPCs, akin to that reported in peripheral blood and spleen HSPCs.^33^ Erythroid bias therefore is a shared feature of steady-state extramedullary HSPCs, perhaps determined by distinct access to environmental oxygen versus the relatively hypoxic environment of the BM.^34^

Our immunofluorescence imaging and spatial transcriptomic studies were essential in confirming the presence of HSPCs in the lung and defining their precise locations and niche. Given the prevailing dogma that hematopoietic precursors widely circulate^35–37^, it was important to rule out that blood contamination was producing our results. We found a few intravascular HSPCs, confirming previous studies, but the vast majority were extravascular and predominately in vascular-rich zones of the lung alveoli. This anatomic location in the lung is similar to the location of HSPCs in the BM, which are closely positioned next to the vascular sinusoids.^38,39^ In the lung, this positioning could be important for seeding of the lung with circulating HSCs and potentially for exiting the lung during hematopoietic stress or during mobilization for stem cell collections. In this niche, it was notable that lung fibroblasts were in close proximity. The lung mesenchyme is well known to be a critical niche supporting epithelial cell development and repair and similar mechanisms could be operable influencing lung HSPCs.^40,41^ Also, we identified a developmental trajectory between lung HSPCs and lung stromal cells. Subpopulations of lung fibroblasts are known to be CD34^+^ and previous lineage-tracing experiments in pulmonary fibrosis have shown a hematopoietic contribution to fibroblasts.^42,43^ Future studies are needed in this area to understand how lung HSPCs could be involved in fibrotic lung diseases.

Our study does have limitations. We used human deceased donors for tissue collection and there may be biases introduced in the mechanisms of their deaths and how that influences HSC biology. There is also donor heterogeneity that could influence our results. Our studies rely heavily on transcriptomics and future studies will need to identify a lung HSC-specific marker to facilitate their identification. We have not addressed the ontogeny of lung HSPCs—something not possible given the restraints of our human tissue study. There is irrefutable evidence that HSCs commonly enter the circulation and microcirculatory beds, and given the functional and molecular similarities of lung HSPCs to other extramedullary HSPCs, this is perhaps the source of the tissue resident HSPCs in the lung.^36,44^ There is precedence, however, for tissue residency to be endowed during development, such as with yolk-sac derived macrophages.^45^ Future studies will be needed to answer this question, including the possibility that hemogenic endothelium in the fetal lung could be the source.^46,47^

Our findings reframe our understanding of the HSPC pool and its molecular diversity and should enable future studies that could potentially lead to therapeutic advances, such as for life-saving HSC transplantation for BM malignancies and failure. In the modern era, transplantation is mainly accomplished using mobilized HSCs obtained from the peripheral blood. We have now shown that this pool is heterogeneous with a sizeable extramedullary component that implies functional heterogeneity given our findings on lung HSC biases. These results have important implications for treatment responses and complications. Our findings may also help to understand the mechanisms of leukemogenesis with the possibility that lung HSPCs are direct targets of environmental carcinogens.^48^ Further, our findings add to our expanding understanding of rare cell types in the lung and their potential functions.^49^

## Supplementary Material

Supplementary Material

## Key points

- Functional hematopoeitic stem cells with unique gene signatures reside in the adult human lung.
- These cells contribute to the pool of hematopoietic stem cells mobilized for stem cell transplantation.
