# Supplementary material for: Decoding functional hematopoietic progenitor cells in the adult human lung
Source: Blood. 2025 Mar 4;145(18):1975–86. doi: 10.1182/blood.2024027884 (PMC7617544; doi:10.1182/blood.2024027884)
Supplement: Supplemental Methods, Tables, Figures, and References [file BLOOD_BLD-2024-027884-mmc1.pdf]

Material and Methods

Human lung, peripheral blood, and bone marrow recovery

Fresh human tissues were obtained from deceased organ donors after either brain death (DBD) or circulatory death (DCD) (Table S1). All patients were on mechanical ventilation and treated in the intensive care unit until organ retrieval. Lungs and vertebral bodies were surgically recovered and immediately placed on ice. Peripheral blood (PB) was collected in sodium heparin tubes. All donor tissues were transported to laboratory via courier from the hospital where the removal procedure took place and immediately processed in the laboratory upon arrival. Depending on procedure and transportation time for each individual donor, the ischemic time for all tissues was kept as short as possible to maximize cellular viability and did not exceed 8 hours.

Isolation of cells from human lungs

Human lung tissue (~5-8g/donor) was rinsed in ice-cold PBS, minced, and placed in digestion medium [300 µg ml^{-1} Liberase^{TM} (Roche) and 10 µg ml^{-1} DNaseI (Roche)] in HBSS (#J67763-AP, Thermo Fisher). Samples were incubated at 37°C for 45 min in a shaker (800 rpm). After the enzymatic digest, the reaction was stopped by adding stop buffer (HBSS, 10% FBS) and the homogenate was filtered through a 100 µm cell strainer (431752, Corning) to obtain a single cell suspension. Cells were pelleted (300 *g*, 5 min) and incubated in red blood cells lysis (RBC) buffer (0.15M NH_4Cl, 10mM $KHCO_3$, 0.1mM EDTA in dH_2O) for 10 min at room temperature. Cells were washed in stop buffer, pelleted again (300 *g*, 5 min, 4°C) and kept at 4°C for the remainder of the protocol.

Isolation of cells from human bone marrow

Vertebral columns of organ donors were split through the intervertebral discs into vertebral bodies (VBs). VBs were crushed along the sagittal axis into small pieces, the vertebral disks were discarded, and the VBs were cleaned from soft tissues. The resulting bone chips were further

rendered into smaller pieces using a bone grinder and placed into Processing medium (phenol-red free RPMI-1640, 25 mM HEPES, 2.5% BSA and 2.5 µg ml^{-1} DNAseI (Roche)) in a 500 ml Nalgene jar. Samples were vortexed for 45 min to allow for passive release of BM cells from the trabecular framework of the VBs. The resulting suspension was filtered through a 180 µm sieve and 100 µm cell strainer to obtain a single cell suspension. Cells were pelleted (300 *g*, 5 min) and incubated in RBC lysis buffer for 10 min at room temperature. Cells were washed in stop buffer, pelleted again (300 *g*, 5 min, 4°C) and kept at 4°C for the remainder of the protocol.

Dead-cell removal and Magnetic Enrichment

Dead-cell removal was performed by gradient centrifugation (800 *g*, 25 min, 4°C) using 12% Iodixanol solution (Optiprep™, Sigma-Aldrich). Live cells were then depleted for lineage-committed cells with biotin antibodies against CD2, CD3, CD11b, CD11c, CD14, CD16, CD19, CD24, CD56, CD66b, CD235, CD31, CD326 and Streptavidin-coupled magnetic beads (130-048-101, Miltenyi) passed through an LS MACS column (Miltenyi, 130-042-401) on a MidiMACS Separator magnet (Miltenyi, 130-042-302). The flow through cells were then designated as lineage-depleted cells. Lineage depleted cells were used in colony formation unit assays or frozen as aliquots of up to $1x10^7$ cells per vial in IMDM, 40% FBS, 15% DMSO and stored at -150°C until use.

MethoCult™ colony forming unit (CFU) assays

Lin-depleted BM ($5x10^3$) or lung ($15x10^3$) cells were plated in duplicate in semi-solid methylcellulose-based MethoCult™ medium (H4434, Stem Cell Technologies) according to the manufacturer's instructions. After incubation for 12-14 days at 37°C in 5% CO_2, hematopoietic colonies were scored based on morphological and phenotypic criteria and quantified by manual counting using brightfield microscopy (Echo, Revolve Microscope). After manual counting, methylcellulose was dissolved, cells were pelleted and stained with antibodies against GlyA/PB,

CD45/APC-Cy7, CD15/APC, CD14/PE, CD41/AF488 (1:400) and LIVE/DEAD Fixable Yellow stain (1:1000) to validate colony composition by flow cytometry.

MegaCult™ CFU assays

Lineage-depleted BM ($5x10^3$) or lung ($15x10^3$) cells were plated in duplicate in collagen-based MegaCult™-C medium (#04960, Stem Cell Technologies) with TPO (50 ng ml^{-1}), IL-3 (10 ng ml^{-1}) and IL-6 (20 ng ml^{-1}). After incubation for 10-12 days at 37°C in 5% CO_2, the colonies were fixed with ice-cold methanol/acetone and stained against CD41 according to the manufacturer's instructions. Thereafter, the colonies were identified based on CD41-immunopositivity and quantified by manual counting using brightfield microscopy (Echo, Revolve Microscope).

Flow cytometry

Clone, supplier and catalogue number for each antibody can be found in the Key Resources Table. After dead-cell removal human lung, blood and BM cells were incubated with FcR block (Biolegend, #422301) to block non-specific binding of antibodies and biotin antibodies against CD2, CD3, CD11b, CD11c, CD14, CD16, CD19, CD24, CD56, CD66b, CD235, CD31, CD326 (1:200) to label lineage-committed cells in MACS buffer (PBS, 0.5% BSA, 2 mM EDTA) for 45 minutes at 4°C. Cells were then pelleted (300 *g*, 5 min, 4°C), washed with MACS buffer and stained for 30 min at 4°C in the dark with antibody panels to phenotype subsets of hematopoietic stem and progenitor cells in MACS buffer. **Panel A:** Streptavidin/BV605, CD34/FITC, CD38/APC, CD45RA/APC-Cy7, CD41/61/PerCP-Cy5.5, CD90/PE, Flt3/BV711, CD49f/Pacific Blue (1:400) and LIVE/DEAD Fixable Yellow stain (1:1000, ThermoFisher #L34959). **Panel B:** Streptavidin/AF647, CD34/FITC (1:400) and LIVE/DEAD Fixable Yellow stain (1:1000). **Panel (C):** For cell cycle analysis, cell surface markers were stained with Streptavidin/BV605, CD34/FITC, CD38/APC, CD45RA/APC-Cy7, CD90/PE, Flt3/BV711 (1:400) and LIVE/DEAD Fixable Yellow stain, followed by fixation in Cytofix/Cytoperm (BD) for 15 min on ice. Cells were

washed in Perm/Wash (BD) and intranuclear staining of Ki-67/PerCP-Cy5.5 (1:400) was performed at 4°C overnight in the dark. DNA content was stained the next day using 1 µg/ml DAPI for 20 min in Perm/Wash at RT. **Panel D:** CD45/AF647, CD45/APC-780, CD33/PerCP, CD19/PB, CD41/FITC, mouse-CD45/PE-Cy7 (1:400) and LIVE/DEAD Fixable Yellow stain (1:1000), **Panel E:** CD45/APC-780, GlyA/PE, GlyA/PB, CD71/PerCP-Cy7, CD41/FITC, mouse-CD45/APC (1:400) and LIVE/DEAD Fixable Yellow stain (1:1000). Prior to flow cytometry, samples were washed and resuspended in 300 µl MACS buffer and filtered through a 100 µm cell strainer. Unstained cells and compensation beads (BD Biosciences) were used for compensation and as controls to set appropriate gates. For flow cytometry analysis, BD LSRII and LSRFortessa cytometers were used and data were analyzed using FlowJo software v9.9 or v10.

Xenotransplantation

For xenotransplantation experiments, 6 week-old triple transgenic female NSG-SGM3 mice (NOD.Cg-Prkdc^{scid} Il2rg^{tm1Wjl} Tg(CMV-IL3, CSF2, KITLG)1Eav/MloySzJ, Stock No: 013062) were purchased at the Jackson Laboratory and housed in a Specific-Pathogen-Free (SPF) animal facility.[1] Mice were allowed to acclimate for 1 week and were 7 weeks old at the time of xenotransplantation. One day before reconstitution, mice were pre-conditioned by sublethal irradiation (2.4 Gy).[2] On the day of transplantation, Lin$^-$ lung and BM cells were thawed and 1.5 x10^6 viable cells were resuspended in 250 µl PBS + 0.1% Pen/Strep (Life Technologies) and intravenously injected into the tail vein of recipient mice.[2] Recipient mice were transplanted with matched lung and BM cells where possible, however, when the number of viable Lin$^-$ cells in the material was not sufficient, additional donors where included. Mice were monitored for signs of GVHD (weight loss ≥5% of maximum body weight) by weekly weighing. 10 weeks post-transplantation, the BM, lung, and blood were investigated for engraftment of human cells. BM cells were isolated from hind limb long bones by centrifugation as previously described[3], lung cells by enzymatic digestion as described above, and 100 µl blood were lysed for 10 min at room

temperature in 1ml RBC buffer. Cells were washed in MACS buffer and stained with antibody panel D and E.

To ensure the detection of low levels of human leukocyte engraftment, samples were stained with two antibodies against different epitopes of hCD45 (cl.30-F11 and cl. HI30). Cells were only considered human leukocytes if they were positive for both CD45 antibodies ($CD45^{++}$) as previously described.[2] Threshold for positive engraftment was set to ≥ 0.01 % $CD45^{++}$ cells of all $CD45^{+}$ (mouse and human) with at least 30 cells recorded in the $CD45^{++}$ gate for BM and lung, and ≥ 15 cells for PB. Cells were determined as myeloid lineage if $CD45^{++}CD33^{+}$ ≥ 20 cells; lymphoid lineage if $CD45^{++}CD19^{+}$ ≥ 20 cells; megakaryocytic lineage if $CD45^{++}CD41^{+}$ ≥ 20 and erythroid lineage if $CD45^{-}$ $GlyA^{+}$ or $CD71^{+}$ ≥ 20 cells. To determine background staining, organs of mice that were irradiated but not transplanted were characterized using the same antibody panel.

Cell sorting for single-cell RNA sequencing

Lineage-depleted cells from the lung and BM were quickly thawed at 37°C and 10 ml of pre-warmed resuspension medium (IMDM, 40% FBS) was drop-wise added. Cells were pelleted at 300 *g* for 5 min, washed with resuspension medium and spun down again. The cell pellet was resuspended in 100 μg ml^{-1} DNaseI (StemCell Technologies) in HBSS (Gibco) and incubated for 15 min at room temperature. Following DNA digest, cells were pelleted, washed in MACS buffer and stained with Antibody Panel B. Stained cells were resuspended in FACS Buffer (1% BSA, 25 mM HEPES, 2 mM EDTA in PBS) at a concentration of $5x10^{6}$ cells/ml, filtered through a 100 μm cell strainer and loaded onto BD FACSAria II cell sorters. Live, Lin^{-} $CD34^{+}$ cells were sorted into 1.5 ml tubes for subsequent 10x Genomics scRNAseq. Live, Lin^{+} cells were collected for demultiplexing the samples from different individuals based on single nucleotide polymorphisms (SNPs).[4]

Single-cell RNA-sequencing using 10x Genomics platform

For each tissue and donor, Live/Lin-/CD34^{+} HSPCs (Table S1) were sorted into 300 μl Sorting Buffer (PBS, 1% BSA, 2 mM EDTA, 25 mM HEPES) and kept on ice until library preparation. Cells were spun down for 5 min at 300 *g* and equal numbers of lung or BM cells from 4 donors were pooled into 2 wells prior to library preparation (Supplemental Figure 6A) and further processed for single-cell sequencing using the ChromiumTM Single Cell 3' Library & Gel Bead Kit v2 (10x Genomics) according to the manufacturer's protocol. Libraries from all donors and tissues were combined prior to sequencing and sequenced on a NovaSeq6000 Sequencing system (SP100).

Data pre-processing, quality control and normalization

The resulting scRNAseq reads were aligned to the human reference genome and Ensembl annotation (GRCh38 genome build, Ensembl annotation version 95) using STAR v2.7.5c[5] with the following parameters: --outFilterType BySJout –outFilterMismatchNoverLmax 0.04 –outFilterMismatchNmax 999 –alignSJDBoverhangMin 1 –outFilterMultimapNmax 1 –alignIntronMin 20 –alignIntronMax 1000000 –alignMatesGapMax 1000000.) and quantified using the CellRanger 3.0.2 suite of tools (https://support.10xgenomics.com). Quality control filters were applied rejecting cells with less than 200 UMI and more than 15% of mithocondrial genes or 50% or ribosomal genes.

The filtered count matrices were normalized, and variance stabilized using negative binomial regression via the scTransform method offered by Seurat.[6,7] To control for confounding variables, we regressed out the effect of cell cycle status, mitochondrial and ribosomal genes. The normalized matrices were reduced to a lower dimension using Principal Component Analyses (PCA) and the first 30 principal coordinates per sample were subjected to a non-linear dimensionality reduction using Uniform Manifold Approximation and Projection (UMAP). Clusters of cells sharing similar transcriptomic signals were initially identified using the Louvain algorithm,

and clustering resolutions varied between 0.6 and 1.2 based on the number and variety of cells obtained in the datasets. Multiplets were estimated using the DoubletFinder package in processed the Seurat objects and subsequently removed.[8] The raw and log-normalized counts per library were then pruned to retain only genes shared by all libraries. Pruned counts matrices were merged into a single Seurat object and the batch (or library) of origin was stored in the metadata of the object. The log-normalized counts were reduced to a lower dimension using PCA and the individual libraries were aligned in the shared PCA space in a batch-aware manner (Each individual library was considered a batch) using the Harmony algorithm.[9] The resulting Harmony components were used to generate batch corrected UMAP visualizations and cell clustering.

Donor genotyping and demultiplexing

To identify SNPs (single nucleotide polymorphisms) that are specific to each donor, Live/Lin$^+$ cells were collected for genotyping. To generate cDNA, the SMART-Seq v4 Ultra Low Input RNA Kit (Takara) was used according to the manufacturer's instructions. Libraries for bulkRNA sequencing then were prepared using Illumina DNA Prep (Illumina) and sequenced on a HiSeqSE50 sequencer. Nucleotide variants were identified from the resulting bam files using the Genome Analysis Tool Kit (GATK, v4.0.11.0) following the best practices for RNA-seq variant calling.[10,11] Libraries containing samples pooled prior to loading were processed using Freemuxlet (https://github.com/statgen/popscle), the genotype-free version of Demuxlet[4] to identify clusters of cells belonging to the same patient via SNP concordance. Briefly, the aligned reads from Cellranger were filtered to retain reads overlapping a high-quality list of SNPs obtained from the 1000 Genomes Consortium (1KG) [10.] Freemuxlet was run on this filtered bam using the 1KG vcf file as a reference, the input amount of samples/pool as a guideline for clustering groups of cells by SNP concordance, and all other default parameters. Cells are classified as singlets arising from a single library, doublets arising from two or more libraries, or as ambiguous cells that cannot be accurately assigned to any existing cluster (due to a lack of sufficient genetic information).

Clusters of cells belonging to a unique sample were mapped to patients using their individual Freemuxlet-generated genotype, and ground truth genotypes per patient identified via bulk RNASeq. The pairwise discordance between inferred and ground-truth genotypes was assessed using the bcftools gtcheck command.[12] Ambiguous, and doublet events were filtered from the major analysis.

Cell clustering and annotation

Clusters of cells sharing similar transcriptomic signal were identified using the Louvain algorithm and loosely grouped into major cell compartments based on cluster signature genes (e.g., hematopoietic progenitor: *AVP, SELL, SPINK2, KLF1, SRGN, GATA1, CA1, HBB, LMO2,* stromal: *PDGFRA, CFD, DCN, APOC, FBLN1, GPC3, COL1A2, COL6A3*, mesothelial: *PRG4, HP, KRT18*). Using the 'SubsetData' command, we generated a new Seurat object including only progenitor cells. Following dimension reduction as described above, clusters of progenitor subsets were detected using the Louvain community analysis to construct the shared nearest neighbor map ('FindClusters'). We identified genes that were consistently expressed across BM and lung-derived cells with the function 'FindConservedMarkers' and assigned cluster identities by comparing cluster-specific marker genes to reference gene expression datasets ([13,14], Supplemental Figure 6). To validate our annotation, we used the command 'find_gene_modules' in Monocle 3[15] to identify modules of co-regulated differentially expressed genes. Gene sets that were specific to certain progenitor subsets were considered signature modules (Supplemental Figure 6) and were compared to the cell type signature gene sets (C8, Human MsigDB Collections) to confirm cluster identity. Pseudotime analysis was performed using the 'orderCells' function in Monocle3 with the cluster 'HSC/MPP' selected as a root for the trajectory.[15]

Differential gene expression

To test differential expression between cells in the HSC/MPP cluster from the lung and BM, the 'FindAllMarkers' function in Seurat using the Wilcoxon rank-sum test was performed. We determined that at least 15% of the cells from each tissue should express the gene. To evalutate the robustness of differentially expressed genes between lung and BM hematopoietic progenitors, Seurat's 'FindMarkers' function was additionally run using 'Bimod' (Likelihood-ratio test for single cell gene expression[16]) and 'MAST' (Model-based analysis of Single-cell Transcriptomics[17]) statistical tests.

Gene set enrichment analysis

Single sample Gene set enrichment analysis (ssGSEA) was performed using the package 'escape'.[18] A gene set score reflecting the degree to which the genes are coordinately up- or downregulated was calculated for each cell. Enrichment was tested for H hallmark, C2 curated, C5 ontology and C8 signature genesets of the MSigDB database 2023.1.[19] The package 'dittoSeq' was used to visualize the results.[20]

Human apheresis donors

Healthy human stem cell donors were treated with a G-CSF mobilization regimen and peripheral blood stem cells were collected by apheresis. We received 1 mL of the mononuclear fraction that would have otherwise been discarded. Apheresis products were kept at 4°C upon collection and processed within 2 hours. The number of cells was determined using an automated Cell Counter (Luna-II™) and cells were cryopreserved as aliquots of 1 x 10^7 live cells per vial in IMDM, 40% FBS and 10% DMSO. Following controlled-rate freezing, cryogenic vials were kept at -80°C for short term storage or transferred to the liquid nitrogen tank to store at -135°C for longer periods until use. On the day of the scRNAseq experiment, cell collections were thawed and processed as described in the section 'Cell sorting for single-cell RNA sequencing'.

Identification of tissue-specific transcriptomic signatures using UCell.

We curated tissue-specific transcriptomic signature panels based on differentially expressed genes between lung and BM HSCs generated in Figure 3 and additionally defined a canonical HSC gene signature (Table S4). To identify these gene signatures in our scRNAseq data of peripheral blood progenitor cells, we employed the package UCell.[21] HSCs were subsetted from the mobilized pool using Seurat's 'subset' function and examined for their expression of canonical, lung and BM HSC signature gene using the function 'ScoreSignatures_UCell'. Based on the distribution of scores across different signatures, summary statistics (minimum, quartiles, median, mean, maximum, and standard deviation) were computed for each signature to establish relevant thresholds. Cells with HSC canonical scores below the specified threshold were removed from the dataset and remaining cells were assigned tissue types (lung or BM) based on their UCell scores. To distinguish cells with distinct signature scores (lung or BM) from cells with almost identical values (lung/BM), we defined a relative difference of >10% as meaningful. All cells that had a canonical HSC score above threshold, but below threshold for either lung or BM signature were classified as 'other' extramedullary sources (i.e., spleen, others).

Identification blood progenitor cells in lung reference data sets

To score gene signatures in the lung reference data sets we used the package 'Ucell'.[21] The lung signature was defined as Lin$^-$, *AVP, SELL, SPINK2, CD63, VIM, MLLT3, SOD2, PLCG2, CD74, MEG3, FTL, CD34, KLF2*, the bone marrow signature as Lin$^-$, *AVP, SELL, SPINK2, CRHBP, HOPX, KLF2, MLLT3, HLA-DRB5, HLA-DRB1, CD34, CD74, SOCS2.* Lin$^-$ genes: *C1QB-, FABP4-, NKG7-, GZMB-, SFTPC-, NAPSA-, LAMP3-, MARCO-, S100A2-, KRT5-, KRT6A-, ACKR1-, VWF-, CD3D-, CLDN5-, DCN-, MGP-, LUM-, AGER-, EMP2-, CAV1-, CAPS-, JCHAIN-, CD79A-, ACTA2-, TAGLN-, SPARCL1-, S100A8-, LYZ-, CD68-, PRR4-, PRB3-, ACKR1-, SPARCL1-, CLU-, DCN-, MGP-, FBLN1-, CCL21-, TFF3-, MMRN1-, ACTA2-, CAPS-, LCN2-, MUC5B-, GRP-, CALCA-, CPVL-, S100B-, TPSAB1-, CPA3-, TPSB2-, CD163-, CD14-, BGN-,*

PDGFRB-, HIGD1B-, HP-, PLA2G2A-, ASCL3-, CD24-, ACTG2-, GPC3-, TEK-, MS4A1-, CD69-, MS4A6A-, APOE-, LAPTM5-, MRC1-, CORO1A-. A Ucell score >0.4 was considered a positive stem cell signature.

Mapping and query annotation

The cells in the integrated lung reference dataset (Human Lung Cell Atlas V2, HLCA V2) with a UCell score >0.4 were projected onto the batch-corrected, Harmony-integrated UMAP of lung and BM hematopoietic progenitor cells (Figure 3A) after finding transfer anchors using the 'MapQuery' function in Seurat. To classify the cells identified in the HLCA V2 based on our UMAP structure (Figure 3A), IDs and prediction scores were calculated using 'TransferData' in Seurat.

Immunofluorescence lung and BM imaging

Tissues were fixed for 3 days in 2% paraformaldehyde. After fixation, vertebral bodies were decalcified in 0.5 M EDTA (pH 7.4) over a week at 4°C under continuous agitation and change of the decalcifying solution every other day. Tissues were washed twice in PBS for 2h and cryoprotected in 30% sucrose in PBS at 4°C overnight. The next day, tissues were embedded face down in OCT medium in disposable plastic beakers (Thermo Fisher Scientific 02–544-30) and frozen as cryostat blocks on dry ice. Lung and BM sections of 100 µm thickness were cut using the block-trimming function of a Leica cryostat cooled to −22°C. Each thick section was quickly transferred into PBS in a 6-well dish, until the OCT dissolved and the lung sections floated. Sections were carefully washed in PBS + 0.3% Triton X-100, then incubated with primary antibodies (1:500, anti-CD34 [Abcam], anti-CD90 [R&D Systems], anti-Lineage [Stem Cell]) in blocking buffer (PBS + 0.3% Triton X-100 + 0.3% BSA + 10% donkey serum) overnight at room temperature. After washing the next day, the floating sections were stained with secondary antibodies (1:500, anti-rabbit AF488; anti-sheep Cy3; anti-Biotin AF647 [Jackson ImmunoResearch]) in PBS and 0.3% Triton X-100 overnight. The wash step was repeated the

next day, and the tissue was again briefly fixed with 1% paraformaldehyde for 5 minutes. The fixative was washed off and the sections were mounted on glass slides using Vectashield mounting medium with DAPI. Images were acquired with a Nikon A1R upright laser scanning confocal microscope with a 25×/1.1 NA Plan Apo LWD water immersion objective using 4 lasers at excitation wavelengths 350 nm, 488 nm, 561 nm and 647 nm.

Molecular Cartography

Tissue sections

Human lung tissue of 4 deceased organ donor samples was frozen in OCT on dry ice and tissue blocks were processed at the facility of Resolve Biosciences, San Jose. Briefly, 10 µm cryosections were prepared and placed within the capture areas of cold Resolve Biosciences slides. Tissue sections then were thawed and fixed according to Molecular Cartography protocol with MF1 for 30 min at 4°C. After fixation, sections were washed twice in 1x PBS for two min, followed by 1 min washes in 70% ethanol at room temperature. Fixed samples underwent an alcoholic series starting with an incubation in isopropanol for 1 min, followed by 95% and 70% ethanol. The samples were used for Molecular Cartography™ (100-plex combinatorial single molecule fluorescence in-situ hybridization) according to the manufacturer's instructions *Day 1: Molecular Preparation Protocol* for human lung, starting with the aspiration of ethanol and the addition of buffer DST1 followed by tissue priming and hybridization. Briefly, tissues were primed for 30 minutes at 37°C followed by 38-48 h hybridization of all probes specific for the target genes (see below for probe design details and target list). Samples were washed the next day to remove excess probes, counterstained with DAPI and fluorescently tagged in a two-step color development process. Regions of interest from each tissue section were chosen and imaged as described below, fluorescent signals removed during decolorization. Color development, imaging and decolorization were repeated for multiple cycles to build a unique combinatorial code for every target gene that was derived from raw images as described below.

Probe Design

The probes for 100 genes were designed using Resolve's proprietary design algorithm. Briefly, the probe-design was performed at the gene-level. For every targeted gene, all full-length protein coding transcript sequences from the ENSEMBL database were used as design targets if the isoform had the GENCODE annotation tag 'basic'.[22,23] For efficiency, the selection of probe sequences was not performed randomly, but limited to sequences with high success rates. To filter highly repetitive regions, the abundance of k-mers was obtained from the background transcriptome using Jellyfish.[24] Every target sequence was scanned once for all k-mers, and those regions with rare k-mers were preferred as seeds for full probe design. A probe candidate was generated by extending a seed sequence until a certain target stability was reached. A set of simple rules was applied to discard sequences that were found experimentally to cause problems. After these fast screens, every kept probe candidate was mapped to the background transcriptome using ThermonucleotideBLAST[25] and probes with stable off-target hits were discarded. Specific probes were then scored based on the number of on-target matches (isoforms), which were weighted by their associated APPRIS level[26] favoring principal isoforms over others. A bonus was added if the binding-site was inside the protein-coding region. From the pool of accepted probes, the final set was composed by picking the highest scoring probes. Table S3 summarizes the gene probes chosen for our study.

Imaging

Samples were imaged on a Zeiss Celldiscoverer 7, using the 50x Plan Apochromat water immersion objective with an NA of 1.2 and the 0.5x magnification changer, resulting in a 25x final magnification. Standard CD7 LED excitation light source, filters, and dichroic mirrors were used together with customized emission filters optimized for detecting specific signals. Excitation time per image was 1000 ms for each channel (DAPI was 10 ms). A z-stack was taken at each region with a distance per z-slice according to the Nyquist-Shannon sampling theorem. The custom CD7

CMOS camera (Zeiss Axiocam Mono 712, 3.45 μm pixel size) was used. For each region, a z-stack per fluorescent color (two colors) was imaged per imaging round. A total of 8 imaging rounds were done for each position, resulting in 32 z-stacks per region. The completely automated imaging process per round (including water immersion generation and precise relocation of regions to image in all three dimensions) was realized by a custom python script using the scripting API of the Zeiss ZEN software (Open application development).

Spot Segmentation

The algorithms for spot segmentation were written in Java and are based on the ImageJ library functionalities. Only the iterative closest point algorithm is written in C++ based on the libpointmatcher library (https://github.com/ethz-asl/libpointmatcher).

Preprocessing

As a first step all images were corrected for background fluorescence. A target value for the allowed number of maxima was determined based upon the area of the slice in μm^2 multiplied by the factor 0.5. This factor was empirically optimized. The brightest maxima per plane were determined, based upon an empirically optimized threshold. The number and location of the respective maxima was stored. This procedure was done for every image slice independently. Maxima that did not have a neighboring maximum in an adjacent slice (called z-group) were excluded. The resulting maxima list was further filtered in an iterative loop by adjusting the allowed thresholds for (Babs-Bback) and (Bperi-Bback) to reach a feature target value (Babs: absolute brightness, Bback: local background, Bperi: background of periphery within 1 pixel). This feature target values were based upon the volume of the 3D-image. Only maxima still in a z group of at least 2 after filtering were passing the filter step. Each z-group was counted as one hit. The members of the z-groups with the highest absolute brightness were used as features and written to a file. They resemble a 3D-point cloud. Final signal segmentation and decoding: To align the

raw data images from different imaging rounds, images had to be corrected. To do so the extracted feature point clouds were used to find the transformation matrices. For this purpose, an iterative closest point cloud algorithm was used to minimize the error between two point-clouds. The point clouds of each round were aligned to the point cloud of round one (reference point cloud). The corresponding point clouds were stored for downstream processes. Based upon the transformation matrices the corresponding images were processed by a rigid transformation using trilinear interpolation. The aligned images were used to create a profile for each pixel consisting of 16 values (16 images from two color channels in 8 imaging rounds). The pixel profiles were filtered for variance from zero normalized by total brightness of all pixels in the profile. Matched pixel profiles with the highest score were assigned as an ID to the pixel. Pixels with neighbors having the same ID were grouped. The pixel groups were filtered by group size, number of direct adjacent pixels in group, number of dimensions with size of two pixels. The local 3D-maxima of the groups were determined as potential final transcript locations. Maxima were filtered by number of maxima in the raw data images where a maximum was expected. Remaining maxima were further evaluated by the fit to the corresponding code. The remaining maxima were written to the results file and considered to resemble transcripts of the corresponding gene. The ratio of signals matching to codes used in the experiment and signals matching to codes not used in the experiment were used as estimation for specificity (false positives).

Cell Segmentation

Cell segmentation was performed using the StarDist algorithm[27] in QuPath[28] to outline individual cell boundaries. A probability threshold of 0.5 was used to determine detections and the cell expansion value used to estimate how far the boundary extends from the nucleus was set to 5μm. Region of interest (ROI) files from the segmentation output were saved and projected on ImageJ along with the detected transcripts.

Expression Matrix Generation

Image analysis was performed in ImageJ using the Polylux tool plugin provided by Resolve BioSciences to examine specific Molecular Cartography™ signals. The QuPath cell-segmentation ROIs representing individual cells were loaded alongside with the gene detection coordinates to generate a cell by gene matrix in which genes and their expression values are represented as columns and cells as rows. The gene CD79A was excluded for downstream analysis due to non-specific staining.

Lung cell annotation

The cell-segmentation ROIs generated in QuPath were loaded into the Data Viewer on the browser-based Resolve Molecular Cartography Platform for visualization and to perform unsupervised clustering (Supplemental Figure 9A-B) Cluster-defining marker genes were used to generate cell-type annotations for the cells present in the sample, a representative example for the analysis steps is provided in Supplemental Figure 9. Samples with few cells and weak staining resulting in failure of clustering were excluded for analysis.

Identification of candidate HSPCs

Due to the rarity of HSPCs in the lung, unsupervised clustering was not suitable to identify candidate cells. To identify putative HSPCs, we therefore used a manual, rule-based approach. We filtered for $CD34^+$ cells and calculated a progenitor-gene enrichment score. Genes in our panel were partitioned into progenitor and non-progenitor genes (Table S3). The progenitor-gene enrichment score was calculated on a per cell basis and is defined as:

$$log_2\left(\frac{\sum Progenitor\ Gene\ Counts}{\sum Non\ Prognitor\ Gene\ Counts}\right)$$

Cells with a score greater than or equal to 1 were considered candidates. Each candidate cell was then visually validated to exclude false positives based on segmentation errors or transcript

location in the cell periphery. After scoring and manual verification, 150 putative HSPCs out of around 14,000 CD34$^+$ cells remained and were each given an annotation corresponding to anatomical location in the lung ('alveolar interstitium', 'peribronchial', 'perivascular', 'intravascular', Figure 4C).

Neighborhood Analysis

In identity celltypes that surround putative HSPCs, simple neighborhoods around each candidate were computed. We defined the neighborhoods as consisting of all cells found within a specified radius of the candidate cell. Distances between cells were calculated using the cell's centroid positions. Neighborhoods of each putative HSPC were aggregated by anatomical locations to obtain anatomically specific neighborhood composition breakdowns.

Co-Occurrence Analysis

To help quantify spatial co-occurrence patterns between cell-types present in the lung and the identified HSPC candidates, we employed a Cluster Co-Occurrence method[29] as described by Tosti et al. and implemented through the co-occurrence function implemented in the SquidPy software package.[30]

The Co-Occurrence score is defined as:

$$\frac{P(clust \mid cond)}{P(clust)}$$

Using the spatial information, the score is calculated by creating a radius of a specified size around each cell present in the dataset and using the encompassed cells for calculation. Thus, given a specified radius, P(clust|cond) is the conditional probability that cluster "clust" is observed given cluster "cond" is observed, while P(clust) is simply the probability that cluster "clust" is observed. Thus, the Co-Occurrence score, in contrast to the simple neighborhood approach

previously described, incorporates information from the entire dataset to more quantitatively capture cluster co-occurrence. Co-occurrence scores conditioned on the presence of HSPC candidate cells (cond = HSPC) were calculated for all lung cell types obtained via clustering (clust = endothelial, epithelial, fibroblasts, macrophages, smooth muscle cells, airway epithelium, lymphoid cells, pericytes, and lymphatic cells). Scores were calculated every 2 μm within an interval of 4 to 30 μm and were subsequently plotted using a rolling average with a window size of 2 to lessen noise while maintaining resolution and shape.

Data and code availability

Sequencing data are deposited in the Gene Expression Omnibus (GEO) with the accession code (*will be provided upon manuscript acceptance*). There are no restrictions on data availability or use. The Human Lung Cell Atlas is publicly available dataset that can be accessed under https://azimuth.hubmap consortium.org/references/human_lung_v2/. Codes used in this study can be found on Github at (*link will be provided upon manuscript acceptance*).

Supplemental Tables Legends

Table S1. Basic demographics and clinical profiles of deceased organ donors.

Table S2. Hematopoietic progenitor subsets and surface marker expression.

Table S3. Molecular probes to characterize cell types in the human lung in spatial transcriptomics. * Probe excluded due to non-specific staining. To simplify color representation, all marker transcripts for each cell type are assigned the same color specified in the table above, unless indicated otherwise.

Table S4. Canonical and tissue-specific HSC gene signatures.

Table S5. Resources Table.

Donor	Gender	Ethnicity	Age	Cause of death	Donation	Comorbidities	Smoking (>20 PY)	Last P/F	Analysis
018	female	White	61	Anoxia	DCD	HTN, CAD	no	263	FC, CFU
019	male	Hispanic	57	ICH	DBD	CAD	no	107	FC, CFU, scSeq
020	male	Hispanic	46	Anoxia	DBD	HTN/HLD, GERD	yes	257	FC, CFU, scSeq
021	female	White	56	ICH	DBD	HTN/HLD, RA, COPD	yes	388	FC, CFU, scSeq
022	female	Asian	31	ICH	DBD	no	no	288	FC, CFU, Xeno
023	male	White	57	Head trauma	DCD	Alcohol Abuse	no	382	FC, CFU, scSeq
024	male	White	45	ICH	DBD	CAD	no	237	FC, CFU, scSeq
025	female	White	47	ICH	DBD	unknown	no	245	FC, CFU, scSeq
026	male	Hispanic	27	Head trauma	DBD	no	no	297	FC, CFU, scSeq, Xeno
027	male	White	30	Head trauma	DBD	no	no	275	scSeq
028	female	Asian	36	Anoxia	DBD	Asthma	no	350	scSeq, Xeno
029	female	White	59	ICH	DBD	HTN, Asthma	no	260	scSeq, Xeno, CFU
030	male	White	43	Anoxia	DCD	Asthma	no	231	FC, Xeno
031	female	White	66	Head trauma	DCD	HTN, Asthma	no	450	FC, CFU
033	male	AI/AN	35	Anoxia	DBD	CAD	no	195	FC, CFU
034	male	White	19	Head trauma	DBD	no	no	275	FC, scSeq
035	male	B/AF	20	Anoxia	DCD	ARVC	no	196	FC, scSeq,CFU SpOmics
036	male	White	32	Anoxia	DBD	Asthma	no	208	SpOmics
037	male	White	39	Head trauma	DBD	no	no	240	FC
038	male	White	53	Anoxia	DBD	no	yes	307	SpOmics
039	male	White	37	Anoxia	DCD	no	no	165	FC
042	female	White	50	Stroke	DBD	no	no	182	SpOmics

Abbreviations:
AI/AN, American Indian or Alaska Native; ARVC, Arrhythmogenic right ventricular cardiomyopathy; B/AF, Black or African American; CAD, Coronary artery disease; COPD, Chronic obstructive lung disease, DBD, Donor after brain death; DCD, Donor after cardiac death; GERD, Gastroesophageal reflux disease; HLD, Hyperlipidemia; HTN, Hypertension; ICH, Intracerebral hemorrhage; P/F, pO_2/FiO_2 ratio; PY, pack years; RA, Rheumatoid arthritis.

FC, Flow Cytometry (Immunphenotyping); CFU, Colony-Forming Unit Assay; scSeq, single-cell RNA Sequencing; SpOmics, spatial Transcriptomics; Xeno, Xenotransplantation.

Table S1: Basic demographics and clinical profiles of deceased organ donors.

**Progenitor cell subset**	**Markers** *(9-10)*
MP (**m**ulti**p**otent cells)	$Lin^{-}CD34^{+}CD38^{-}CD45RA^{-}$
HPC (**h**ematopoietic **p**rogenitor **c**ell)	$Lin^{-}CD34^{+}CD38^{+}$
Hematopoietic Stem Cell (HSC)	$Lin^{-}CD34^{+}CD38^{-}CD45RA^{-}CD90^{+}(CD49f^{+})$
Multipotent Progenitor (MPP)	$Lin^{-}CD34^{+}CD38^{-}CD45RA^{-}CD90^{-}(CD49f^{-})$
Multipotent Lymphoid Progenitor (CLP)	$Lin^{-}CD34^{+}CD38^{-}CD45RA^{+}CD90^{-}$
Common Myeloid Progenitor (CMP)	$Lin^{-}CD34^{+}CD38^{+}CD45RA^{-}Flt3^{+}$
Megakaryocyte-Erythroid Progenitor (MEP)	$Lin^{-}CD34^{+}CD38^{lo/+}CD45RA^{-}Flt3^{-}$
Colony-Forming Unit-Megakaryocyte (CFU-MK)	$Lin^{-}CD34^{+}CD45RA^{-}Flt3^{-}CD41^{+}$
Granulocyte-Macrophage Progenitor (GMP)	$Lin^{-}CD34^{+}CD38^{+}CD45RA^{+}Flt3^{+}$

Table S2: Hematopoietic progenitor subsets and surface marker expression.

Cell type	Markers
AT1 cell	AGER, CAV1, RTKN2
AT2 cell	SFTPC, SFTPD, LAMP3
Fibroblast	CLU, FBLN1, SERPINF1, PDGFRA, CFD, GPC3, COL1A2
Pericyte	PDGFRB, PTN, HIGD1B
Lymphatic	CCL21, TFF3, MMRN1
Smooth Muscle	MYH11, ACTA2, ACTG2
Capillary	VWF, PECAM1, TEK, (CD34)
Artery	GJA5, HEY1, DKK2
Vein	ACKR1, CPE
Mesothelial	HP, CALB2
Leukocyte	PTPRC
Macrophage	MARCO, C1QB, FABP4
Monocyte	VCAN, CD14, FCGR3A
Neutrophil	CSF3R, G0S2, FCGR3B
T cell	CD3D, IL7R, CD4 or CD8A
B cell	BANK1, MS4A1, CD79A*
DC	IRF7, LILRA4, PLD4
NK cell	PRF1, NKG7, GZMB
MK/ Platelet	ITGA2B, PPBP, PF4
Mast cell	HPGDS, CPA3
Niche factors	CXCR4, CXCL12, KITLG, THPO
CD34⁺	CD34
Progenitor	SPINK2, SELL, HOPX, CRHBP, HBB, KLF1, BLVRB, CA1, HBD, FCER1A, THY1, CD38, ITGA6, MPL, KIT, MEG3, FCER1A, PCLAF, AHSA1, BTF3, CHCHD2, TMPO, ANKRD12, LAPTM5, TUBB, NUCKS1, TUBA1B, CENPF, RORA, FOSB, GSTM5, HMGB2

Table S3: Molecular probes to characterize cell types in the human lung in spatial transcriptomics. * Probe excluded due to non-specific staining. To simplify color representation, all marker transcripts for each cell type are assigned the same color specified in the table above, unless indicated otherwise.

Gene signatures

canonical	lung	BM
AVP	HSPA1A	FCER1A
SELL	MTRNR2L12	HLA-DRB5
CD34	PLCG2	HLA-DRB1
MLLT3	SERPING1	SPINK2
CRHBP	GSTM5	H1FX
	MEG3	FAM30A
	CD63	HOPX
	SOD2	CAT
	CEBPB	VAMP8
	NFE2L2	CD74
	VIM	MYB
	IL6ST	GIHCG
	TFPI	ARMH1
	RRBP1	IGLL1
	CYBRD1	ILF3-DT

Table S4: Canonical and tissue-specific HSC gene signatures.

Flow Cytometry		SOURCE	IDENTIFIER
**Antibodies**	**Host**		
anti-human CD2 Biotin (clone RPA-2.10)	mouse	StemCell	Cat #60007BT
anti-human CD11b Biotin (clone ICRF44)	mouse	StemCell	Cat #60040BT
anti-human CD3 Biotin (clone UCHT1)	mouse	StemCell	Cat #60011BT
anti-human CD66b Biotin (clone G10F5)	mouse	BioLegend	Cat #305120
anti-human CD16 Biotin (clone 3G8)	mouse	StemCell	Cat #60041BT
anti-human CD11c Biotin (clone 3.9)	mouse	Invitrogen	Cat #11-0116-82
anti-human CD14 Biotin (clone 6103)	mouse	Invitrogen	Cat #13-0149-82
anti-human CD19 Biotin (clone HIB19)	mouse	StemCell	Cat #60005BT
anti-human CD235 Biotin (clone HIR2)	mouse	BioLegend	Cat #306618
anti-human CD56 Biotin (clone HCD56)	mouse	StemCell	Cat #60021BT
anti-human CD24 Biotin (eBioSN3)	mouse	Invitrogen	Cat #13-0247-82
anti-human CD31 Biotin (WM-59)	mouse	Invitrogen	Cat #13-0319-82
anti-human CD326 Biotin (1B7)	mouse	Invitrogen	Cat #13-9326-82
anti-human CD140a Biotin (16A1)	mouse	Biolegend	Cat #323503
anti-human CD34 FITC (clone 581)	mouse	StemCell	Cat #60013FI
anti-human CD38 APC (clone HIT2)	mouse	Invitrogen	Cat #17-0389-42
anti-human CD41/61 PerCP-Cy5.5 (clone A2A9/6)	mouse	BioLegend	Cat #359813
anti-human CD45RA APC-Cy7 (clone HI100)	mouse	BD Pharmingen	Cat #560674
anti-human CD49f Pacific Blue (clone GoH3)	rat	BD Pharmingen	Cat #60037PB.1
anti-human CD90 PE (clone 5E10)	mouse	StemCell	Cat #60045PE.1
anti-human CD135 BV711 (clone 4G8)	mouse	BD Horizon	Cat #563908
anti-human CD14 PE (clone 61D3)	mouse	Invitrogen	Cat #12-0149-41
anti-human CD15 APC (clone 4G8)	mouse	Invitrogen	Cat #17-0158-41
anti-human CD45 APC-Cy7 (clone 2D1)	mouse	Biolegend	Cat #368515
anti-human CD45 APC-eFluor 780 (clone 30-F11)	mouse	Invitrogen	Cat # 47-0451-82
anti-human CD45 AF647 (clone HI30)	mouse	Biolegend	Cat # 304056
anti-human GlyA Pacific Blue (clone HI264)	mouse	Invitrogen	Cat #349107
anti-human GlyA PE (clone HIR2)	mouse	Invitrogen	Cat #12-9987-80
anti-human CD41a AF488 (clone HIP8)	mouse	BioLegend	Cat #303723
anti-human CD41a FITC (clone HIP8)	mouse	BD Pharmingen	Cat # 555466
anti-human Ki-67 PerCP-Cy5.5	mouse	BioLegend	Cat #350519
anti-human CD33 PerCP (clone WM2)	mouse	Invitrogen	Cat # 50-113-7770
anti-human CD33 PE (Clone 6C5/2)	mouse	R&D Systems	Cat # FAB1137P
anti-human CD71 PerCP-Cy5.5 (clone CY1G4)	mouse	BioLegend	Cat # 334113
anti-human CD3 BB700 (clone SK7)	mouse	BD Horizon	Cat # 566575
anti-human CD19 BB700 (clone SJ25C1)	mouse	BD Horizon	Cat # 566396
anti-human CD19 PB (clone LT19)	mouse	BioRad	Cat # MCA1940PBT
anti-human CD19 PE-Cy7 (clone HIB19)	mouse	NovusBio	Cat # NBP1-42967

anti-mouse CD45 PE-Cy7 (clone 30-F11)	rat	BD Biosciences	Cat # 552828
anti-mouse CD45 APC (clone 30-F11)	rat	BioLegend	Cat # 103112
anti-mouse CD45 APC-Cy7 (clone 30-F11)	rat	BioLegend	Cat # 557659
Streptavidin-BV605		BioLegend	Cat #405229
Streptavidin-AF647		BioLegend	Cat # 405237
LIVE/DEAD Fixable Yellow Stain		ThermoFisher	Cat #L34959
Human TruStain FcX^{TM} (Fc Blocker)		BioLegend	Cat #422302
TruStain FcX™ (anti-mouse CD16/32) Antibody		BioLegend	Cat # 101319

**Immunhistochemistry**		**SOURCE**	**IDENTIFIER**
**Antibodies**	**Host**		
anti-human CD34 (clone EPR2999)	rabbit	Abcam	Cat #ab110643
anti-human CD90	sheep	R&D Systems	Cat #AF2067
anti-human CD45 (clone D9M8I)	rabbit	CellSignalling	Cat # 13917S
anti-human GlyA (clone EPR8200)	rabbit	Abcam	Cat #ab129024
anti-rabbit IgG (HRP)	donkey	Abcam	Cat #ab98493
Alexa Fluor 647 anti-Biotin	mouse	Jackson Immuno	AB_2339046
Alexa Fluor 488 anti-rabbit	donkey	Jackson Immuno	AB_2313584
Cy3 anti-sheep	donkey	Jackson Immuno	AB_2315778

**Experimental models**		
**Organisms/strains**	**SUPPLIER**	**IDENTIFIER**
NOD.Cg-Prkdc^{scid} Il2rg^{tm1Wjl} Tg(CMV-IL3, CSF2, KITLG)1Eav/MloySzJ	Jackson Laboratories	Strain #:01306

**Critical commercial assays**		
**Assay**	**SUPPLIER**	**IDENTIFIER**
MethoCult^{TM} H4100	StemCell Technologies	Cat #04100
MegaCult™-C Complete Kit with Cytokines	StemCell Technologies	Cat #04971

**Software and Algorithms**		
**Software**	**SUPPLIER**	**IDENTIFIER**
FlowJo Software	Becton, Dickinson & Company	Version 9.9/ 10
Matlab	The MathWorks, Inc.	R2023b
GraphPad Prism	GraphPad Software	Version 10.0.2
R/ RStudio	https://www.r-project.org	Version 4.0.3
Python	Python Software Foundation	Version 3.12.1
ImageJ	NIH	Version 1.53k
Molecular Cartography, Recognize	Resolve Biosciences	Version 1.3.6-8

**Software and Algorithms**		
**Algorithms**	**SUPPLIER**	**IDENTIFIER**
STAR	Dobin, A. et al. (2013)[5]	Version 2.7.5c
CellRanger	https://support.10xgenomics.com	Version 3.0.2
Seurat	Hafemeister C. et al. (2019)[6],<br>Hao et al. (2021)[7]	Version 4.3.0
DoubletFinder	McGinnis C.S. et al. (2019)[8]	Version 2.0
Harmony	Korsunsky et al. (2019)[9]	Version 0.1
Genome Analysis Tool Kit	DePristo M.A. et al. (2011)[11],<br>Van der Auwera G.A. et al. (2013)[10]	Version 4.0.11.0
Freemuxlet	Kang H.M. et al. (2018)[4]<br>https://github.com/statgen/popscle	Version 2.0.1
Monocle 3	Cao J. et al. (2019)[15]	Version 1.3.1
Escape	Borcherding N. et al. (2021)[18]	Version 1.12.0
DittoSeq	Bunis et al. (2021)[20]	Version 3.18
UCell	Andreatta M. et al. (2021)[21]	Version 2.5
StarDist	Schmidt U. et al. (2018)[27]	Version 0.8.5
QuPath	Tosti L. et al. (2021)[29]	Version 0.5.0
Squidpy	Palla G et al. (2022)[30]	Version 1.3.1

Table S5: Resources Table.

Supplemental Figure Legends

Supplemental Figure 1. Numbers of hematopoietic progenitor cells in the lung and PB and association of HSPC frequency with age and gender. (A) Representative human lungs and sampling locations. Patients with no significant lung pathologies or hematological disorders of any age, gender and ethnicity were selected for our study. Lungs were inspected for visible injury and tissue was collected from normal-appearing regions (white circles). **(B)** The absolute numbers of immunophenotypic hematopoietic progenitors were quantified in 1 mL of peripheral blood (red) and 1 gram of lung tissue (grey) by flow cytometry (n=8). Cell numbers per cm^3 for each tissue were calculated based on published densities (https://www.aqua-calc.com/calculate/weight-to-volume) for blood (blue, Ref ID 362, 1.0565 g/cm^3) and for lung (white, Ref ID 1762, 1.050 g/cm^3). Bars indicate mean ± SD, dot colors represent individual donors, Student's t-test $^*p< 0.05$; $^{**}p<0.01$; $^{***}p<0.001$. **(C)** Scatterplot illustrating the correlation between age and HSC/MPP cell frequency (left panel) in the BM (blue) and lung (red). The regression line visualizes the association of the variable, Pearson's correlation coefficient r and associated p-values (p) are indicated. Correlation between age and HPC frequency (right panel) in the BM and lung, respectively. **(D)** Box and whisker plot with individual values showing the MP and HPC frequencies separated by donor sex (male, grey; female, green). ANOVA followed by Sidak's multiple comparison test. ns, not significant.

Supplemental Figure 2. Lineage panel modification to reduce fibroblast capture. (A) Published datasets[14] as well as our own scRNA-seq data suggest that platelet-derived growth factor receptor alpha (PDGFRα) could mark most fibroblasts in the lung. To test this, an antibody against PDGFRα was added to the lineage panel (Panel II) and the results were compared to the lineage panel without PDGFRα (Panel I). **(B)** Representative flow cytometry plots of lung cells show the impact of the PDGFRα antibody on the Live/Lin^-/$CD34^+$ cell population. 'Control' cells

were stained with the viability dye only to determine autofluorescence. **(C)** Frequencies of immunophenotypes as percentage of Live/Lin$^-$/CD34$^+$ cells with Panel I versus II; no significant differences were detected across 6 donors (ANOVA followed by Sidak's multiple comparison test.)

Supplemental Figure 3. Evaluation of lineage surface marker expression in MethoCult^{TM} colonies using flow cytometry. (A) Representative colonies of matched lung and BM Lin$^-$ cells, and PB nucleated cells from one organ donor. Scale Bar, 200 µm. **(B)** Following visual colony identification at 14 days of culture, MethoCult™ media was dissolved to generate a single cell suspension for flow cytometric analysis. **(C)** Representative flow plots of lineage marker expression on BM and lung colonies (GlyA, erythroid; CD45, leukocytic; CD14, monocytic; CD15, neutrophilic; CD41, megakaryocytic). **(D)** Cellular composition of colonies across 8 matched donors, marker expression given as percentage of single, live cells (%). Student's t-test *$p<0.05$; ns, not significant. **(E)** Bar graphs representing mean absolute colony counts with standard deviation per 15*10^3 Lin$^-$ cells from the BM (black, n = 8 donors) and lung (blue, n = 8 donors), and per 50*10^3 nucleated cells for PB (red, n = 7 donors). Individual dots indicate replicates. Note that the colony counts of PB are not directly comparable to those of the BM and lung due to different cell inputs. **(F)** Culture initiating capacity of 50*10^3 nucleated cells from peripheral blood in MethoCult^{TM} (n=7). CFU, colony-forming unit; BFU-E (purple), burst-forming unit-erythroid; G (orange), granulocyte; M (red), macrophage; GM (pink), granulocyte macrophage; GEMM (black), granulocyte, erythroid, macrophage, megakaryocyte. **(G)** Cellular composition of PB colonies measured by flow cytometry across 7 donors, marker expression given as percentage of single, live cells (%).

Supplemental Figure 4. Colony formation of immunophenotypic HSCs and HPCs from the lung and BM in MegaCult^{TM}. (A) Sorted HSC fractions (Live/Lin$^-$/CD34$^+$/CD38$^-$/CD45RA$^-$/CD90$^+$)

from the BM and lung were cultured in MegaCult^{TM}. Representative colonies at day 10 (d10) of colony formation in the collagen-gel observed under phase-contrast (PC) microscopy and fixed colonies stained against CD41 (scale bar, 25 µm). Colony size and quantity for HSCs from the BM and lung. **(B)** Colony formation of the HPC fraction, respectively. Representative fixed colonies colonies stained against CD41 (scale bar, 100 µm). Colony size and quantity for HPCs from the BM and lung. Stacked bars represent mean proportion ± SD, Kruskal-Wallis test, ns, not significant; ****p<0.0001. Bar graph represents mean number of colonies ± SD, Student's t-test, *p<0.03. Colors of the dots represent individual donors.

Supplemental Figure 5. **Engraftment efficiency of human cells in the BM, lung and PB of recipient mice after xenotransplantation of HSPCs from BM or lung. (A)** Engraftment efficiency of human cells in the BM, lung and PB of recipient mice after xenotransplantation of HSPCs from either the human BM or lung (n=7 mice/group, % relative to CD45$^+$ cells). Fisher's exact test; ns, not significant. **(B)** Human erythroid cell expansion (CD45$^-$, hGlyA$^+$ or hCD71$^+$) in the BM, lung and PB of recipient mice after xenotransplantation with HSPCs from either the human BM or lung (n= 7/group, % relative to CD45$^-$ cells). Fisher's exact test, ns, not significant. **(C-E)** Lineage expansion of human lymphoid (CD45^{++}CD19$^+$), human myeloid (hCD45^{++}CD19$^+$), human megakaryocytic (CD45^{++}CD41$^+$) and human erythroid (CD45$^-$GlyA$^+$CD71$^+$) cells as percentage of all human cells in the BM (**C**), lung (**D**), and PB (**E**) of recipient mice. Mean ± SD; individual data points for each animal are plotted as gray dots. Student's t-test, p-values are given in the graph.

Supplemental Figure 6. Pipeline for multiplexed scRNAseq of Lin$^-$CD34$^+$ cells from matched lung and BM and annotation of hematopoietic progenitor subsets. (A) Single-cell suspensions generated from lung and BM were lineage-depleted (Lin$^-$ cells) and cryopreserved. For each experimental batch (4 donors), Live/Lin$^+$/CD34$^+$ cells were flow sorted and encapsulated

into 2 GEMs (Chip, GEM wells). 10x Chromium^{TM} Single Cell 3' v2 libraries were prepared, pooled and sequenced. Live/Lin^{+} cells were collected for bulk RNA-sequencing and subsequent SNP calling for donor demultiplexing. The experiment was carried out in 3 batches (3x). **(B)** Sorting strategy to collect Lin^{-}CD34^{+} cells for scRNAseq. (**C**) Batch-corrected UMAP with major cell compartments based on cluster marker genes. **(D)** Proportion of progenitor, stromal and mesothelial cells in sorted Lin^{-}CD34^{+} from the BM and lung. **(E)** scRNAseq quality control metrics showing gene (nGene) and UMI (nUMI) counts per cell for the progenitor, stromal and mesothelial compartment in the BM and lung. **(F)** Left panel: UMAP representation and Louvain clustering (resolution 0.5) of the progenitor subset for BM and lung. Right panel: overview of marker genes associated with each cluster based on Seurat's 'FindConservedMarkers' function. **(G)** Dotplot representing the expression levels of conserved marker genes.

Supplemental Figure 7. HSC-specific gene modules and gene expression profiles. (A) Aggregate module scores generated with Monocle3's 'find_gene_modules' function grouping similar patterns of gene expression within the HSC cluster. Genes co-regulated in the module highly specific for HSCs (Module 54) are noted below. **(B)** Expression values of selected HSC-associated genes within the module.

Supplemental Figure 8. Identification of HSC signatures in published scRNA-seq datasets of the human lung. Due to their rarity, HSCs might be masked by the noise of other highly abundant cell types in the lung. **(A)** UMAP representation with annotations of the lung reference data set (https://azimuth.hubmapconsortium.org/references/human_lung_v2/) representing 584,944 cells from 9 datasets[14,31-38]. **(B)** CD34 expression levels across all lung cell entities in the reference dataset. **(C, D)** Ucell gene signature scoring to identify putative HSCs based on their gene expression profile in the lung. **(C)** UMAP projection highlighting cells with a Ucell score for lung HSC signatures >0.4. **(D)** UMAP projection highlighting cells with a Ucell score for BM HSC

signatures >0.4. **(E)** Using Ucell signature scoring, we identified 120 putative HSCs in the human cell lung atlas V2 (HCLA V2). These cells were projected on the UMAP structure of hematopoietic progenitors from the lung and BM generated in Figure 3A. Predicted IDs of the putative HSCs are shown in the legend. **(f)** Pie graph showing the proportion of predicted cell identities across the 120 putative HSCs in the HLCA V2 categorized by UCell scoring. Cell counts are indicated in parentheses. **(G)** Representation of cells projecting on the HSC/MPP cluster (n= 43) in individual samples from datasets within the integrated lung reference atlas. Out of 584,944 cells in the HLCA V2, 43 HSC/MPPs were identified representing a frequency of 0.007%.

Supplemental Figure 9. Immunofluorescence imaging of putative HSCs in the human lung and BM. (A) Thick sections (100 µm) of the human lung and decalcified VBs were stained with antibodies against CD34 (FITC; green), CD90 (PE; red), and lineage markers (AF647; magenta). $Lin^{-}/CD34^{+}/CD90^{+}$ cells were considered putative HSCs. Representative cells for the lung (n=3) and BM (n=1) are shown. **(B)** 3D reconstruction and 2D plane for putative HSC ($Lin^{-}/CD34^{+}/CD90^{+}$) in the human lung.

Supplemental Figure 10. Pipeline to identify putative HSPCs and delineate neighboring cells. (A) Mapping of target transcripts at subcellular resolution using the Molecular Cartography platform by Resolve. Cell segmentation was performed using DAPI-based cell detection and adapting the QuPath algorithm for lung tissue. The generated cell segmentation ROI sets were used to compute cell type clustering. **(B)** Cells were clustered into distinct populations based on their marker gene expression and annotated through comparison with reference gene sets. Dotplot representing the expression levels of lung cell marker genes. UMAP visualization pseudo-colored by annotated lung cell types. **(C)** Heatmap representation of marker gene expression values normalized for each gene. Cell segmentation ROIs have been sorted by assigned cell type cluster, indicated by the color in the top row and in **(B)**. Bar graph illustrating the number of cells

assigned to a particular cluster in the lung tissue shown in **(A)**. **(D)** Pseudocoloring of **(A)** based on the clustering results in **(B)**. To identify putative HSPCs in the lung, a progenitor-gene enrichment score was calculated. **(E)** Cells positive for CD34 (blue) and low lineage transcript count with a high $Score_{prog}$ were considered candidates ($Score_{prog}$ >1; indicated by the dotted line). All candidates were validated visually (validated candidate HSPCs; magenta) to exclude false positives due to segmentation errors. **(F)** Validated candidate HSPCs (magenta) projected on the segmented lung tissue section in **(A)**.

Supplemental Figure 11. Gene expression of putative HSPCs across all lung tissue sections. **(A)** Representative examples of validated candidate HSPCs in the human lung. **(B)** Heatmap displaying gene expression normalized per cell of putative HSPCs across all lung tissue sections. **(C)** Number of marker transcripts within a radius of 20µm from putative HSPCs across all anatomic locations.

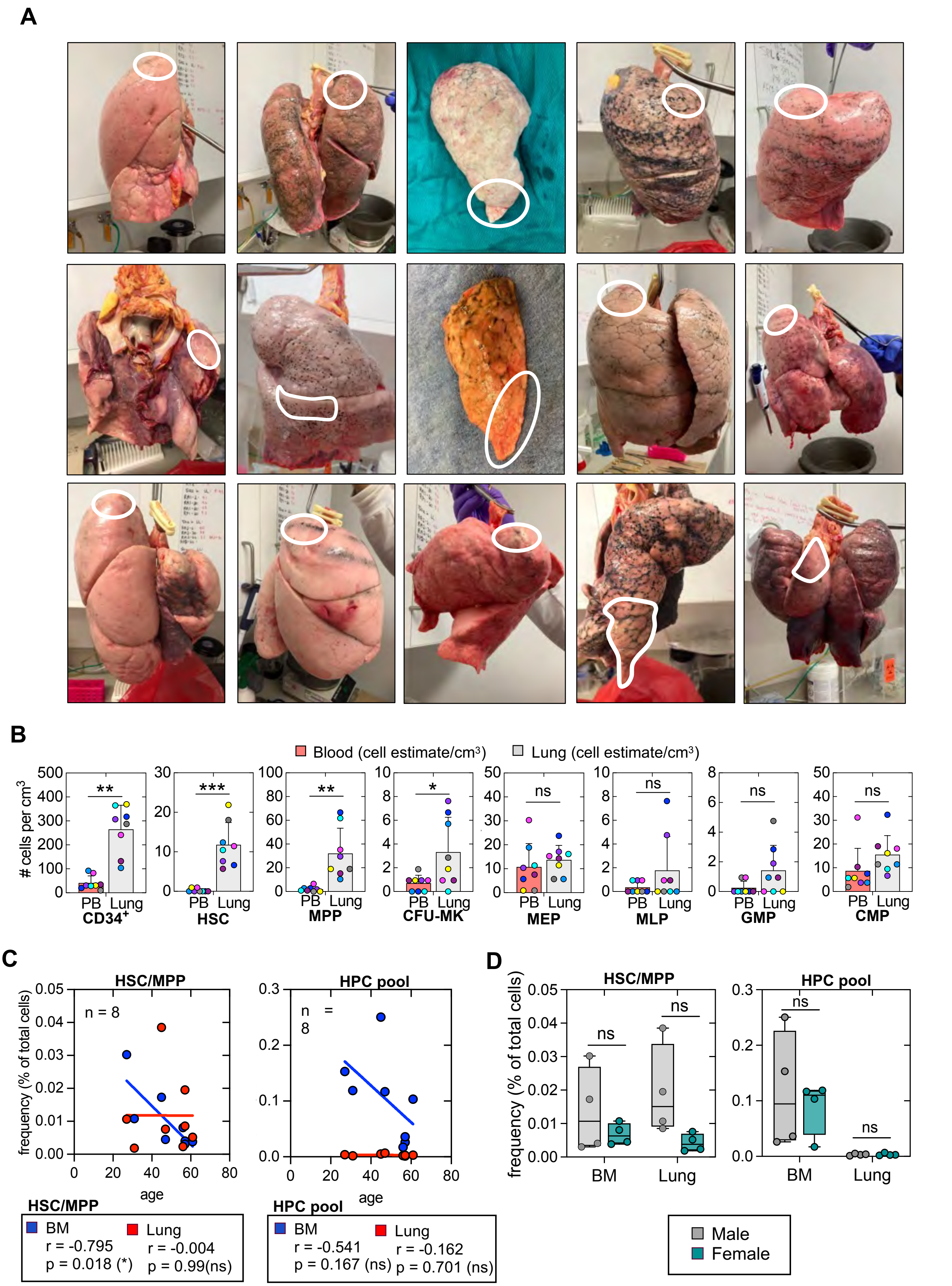

Supplemental Figure 1

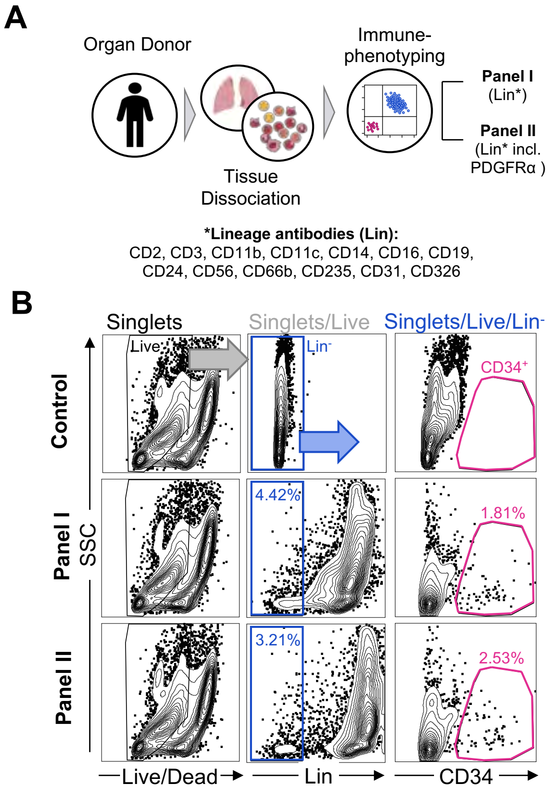

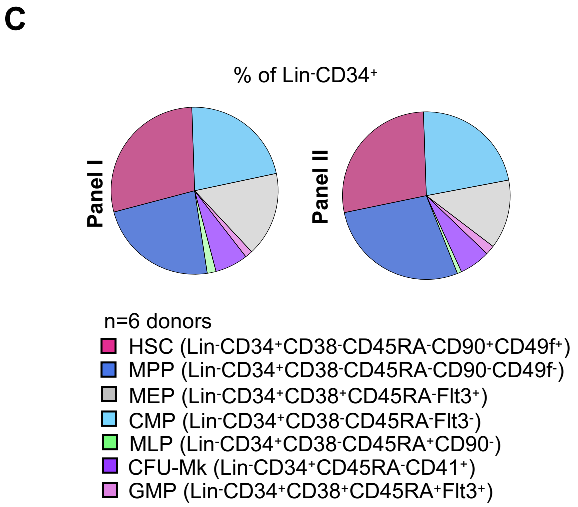

Immunophenotype (% of Lin^-CD34^+)	**Panel I**	**Panel II**	**p-value**
HSC	27.1 ± 12.3	26.5 ± 15.4	0.84 (ns)
MPP	24.6 ± 8.9	28.6 ± 12.8	0.28 (ns)
MEP	16.0 ± 6.3	13.7 ± 7.8	0.34 (ns)
CMP	22.6 ± 7.4	22.5 ± 12.9	0.95 (ns)
MLP	1.8 ± 2.3	0.9 ± 0.7	0.23 (ns)
CFU-Mk	6.3 ± 3.2	6.0 ± 2.6	0.67 (ns)
GMP	1.6 ± 1.1	1.8 ± 2.3	0.64 (ns)

Supplemental Figure 2

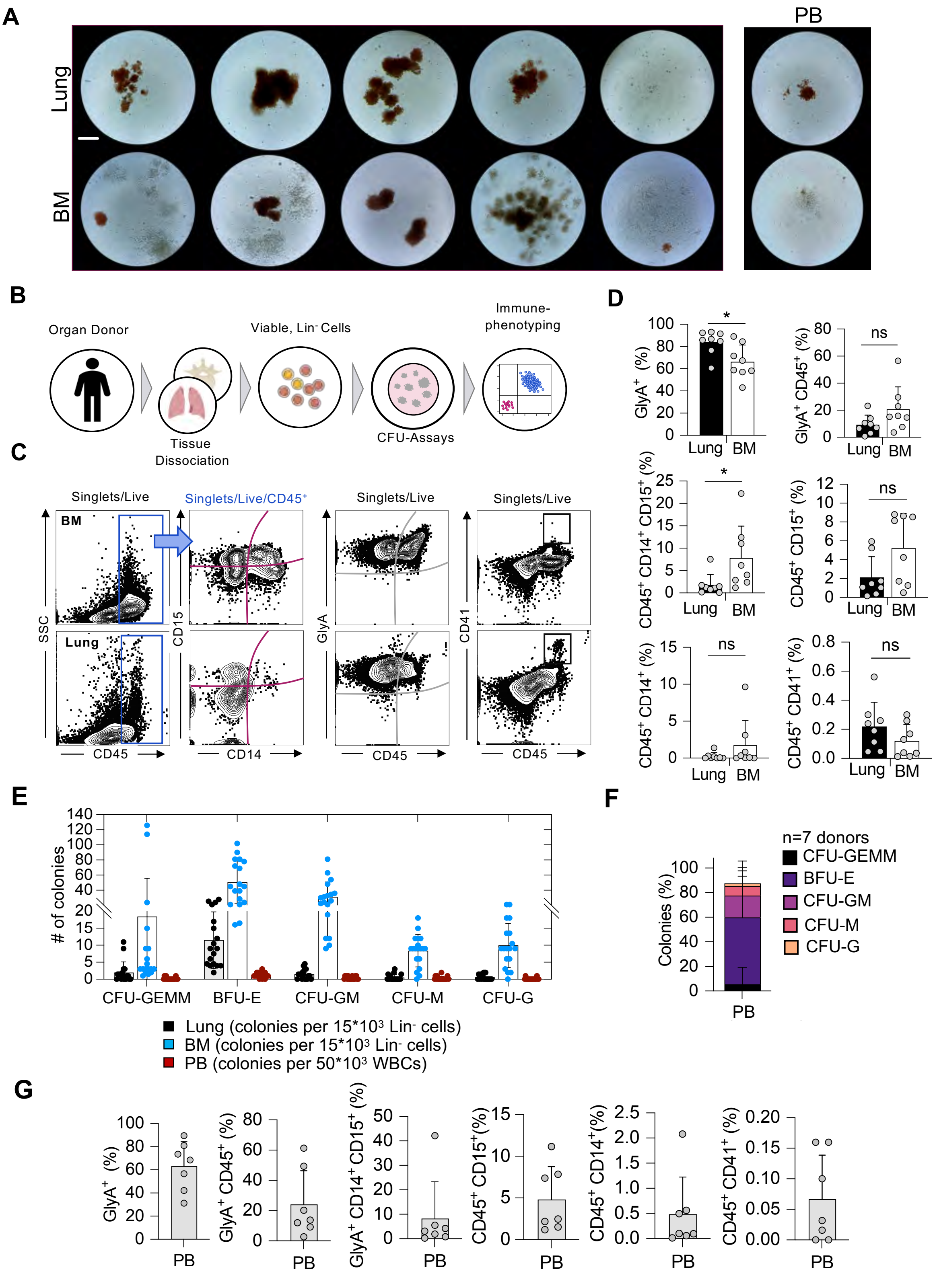

Supplemental Figure 3

A

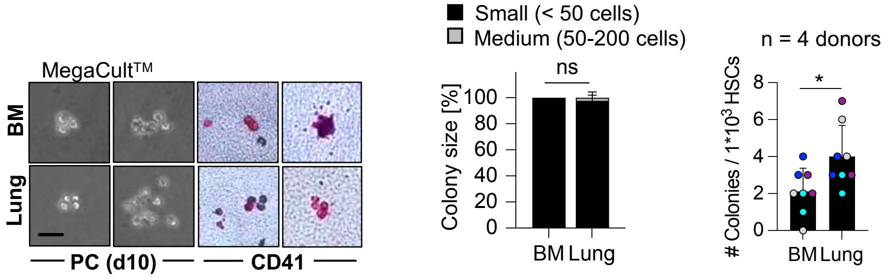

B

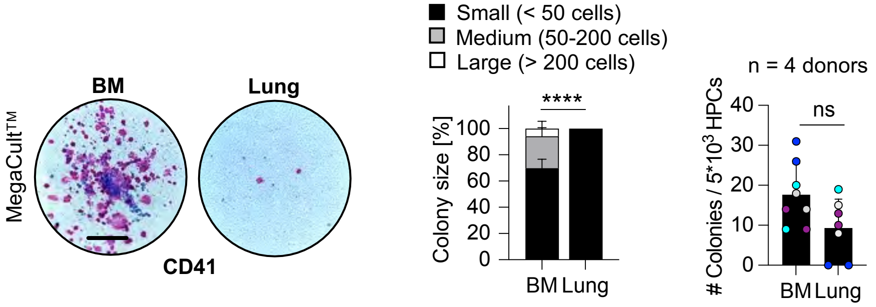

Supplemental Figure 4

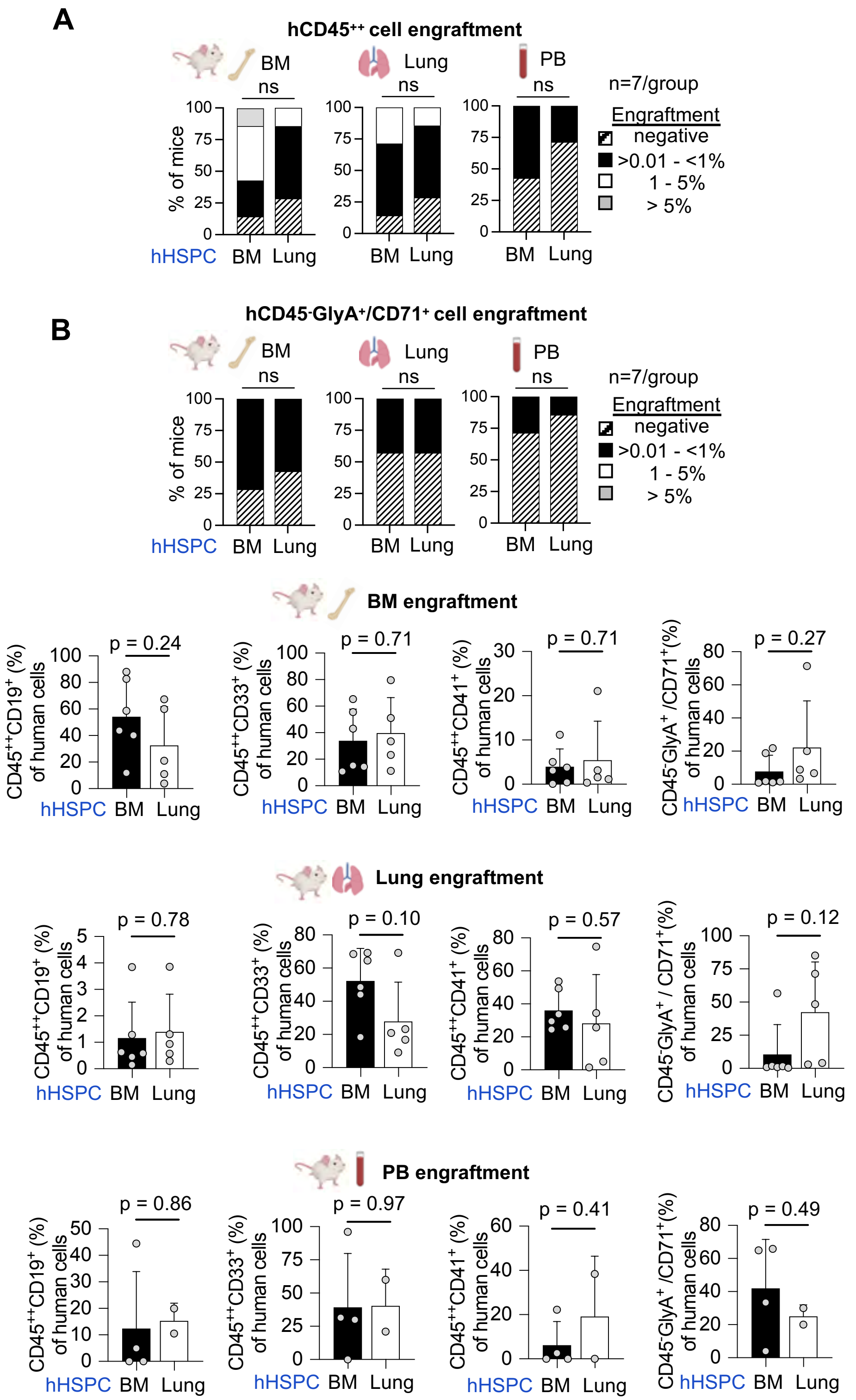

Supplemental Figure 5

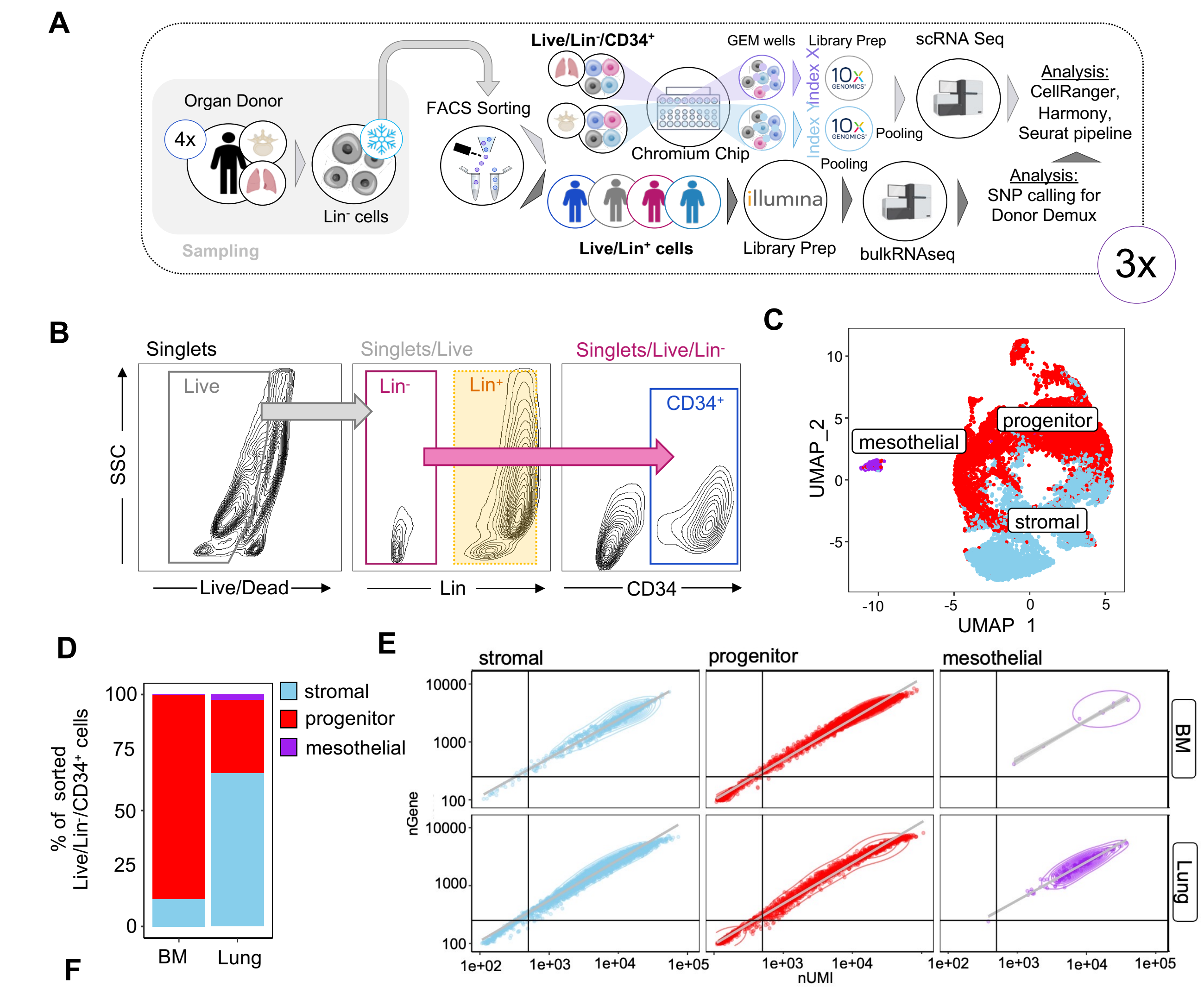

F

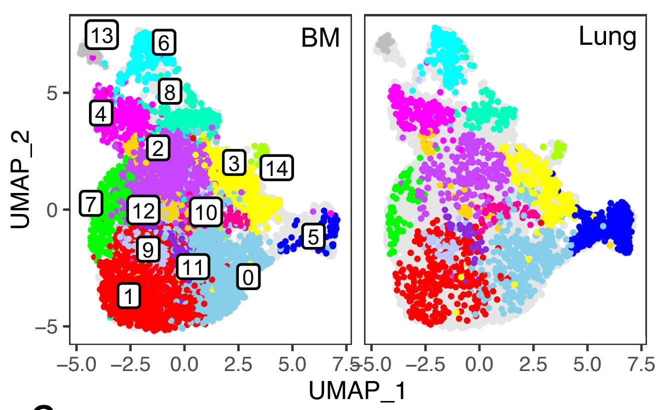

Cluster	Progenitor subset	Conserved Marker genes
0	Erythroid	HBB, GLRX5, SOD1
1	lateERY	HBD, CA1, HBB, HMGB2, STMN1, DUT
2	MPP/multiLin	LEPROT, CELF2, PNRC1, YPEL3, CCNI, DUSP1
3	Stromal/prog mix	APOC1, CTSH, VIM, APOE, PDLXDC2
4	BaEoMa	TPSB2, CD69, CPA3, HPGDS, GATA2
5	Stromal	APOD, DCN, GPX3, CFD, COL6A2
6	HSC	AVP, CD74, SPINK2, SELL, MLLT3, HOPX
7	EMP	PLEK, GP1BB, HBD, ITGA2B, CD38, HEMGN
8	MPP	SOX4, KLF2, JUN, JUNB, FOSB, BTG2
9	Cycling cells	TUBA1A, TUBB, STMN1, BIRC5, TOP2A
10	Not specific	CCNL1, FOSB, NEAT1, NKTR
11	earlyERY	CA1, CA2, HBD, FAM178B, KLF1, BLVRB
12	Not specific	no markers
13	My	LYZ, TYROBP, FCER1G, S100A8, CD14
14	ND	no markers

G

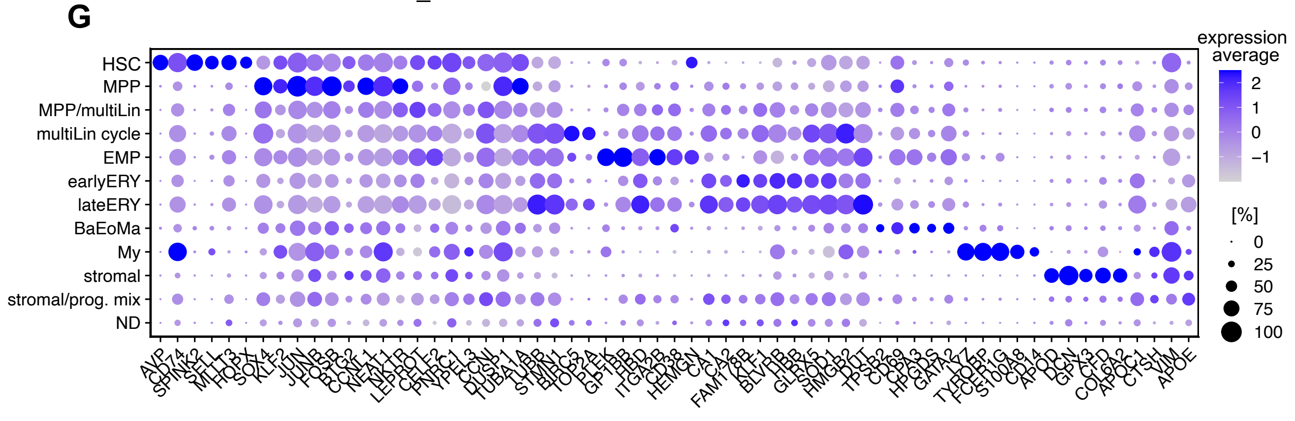

Supplemental Figure 6

A

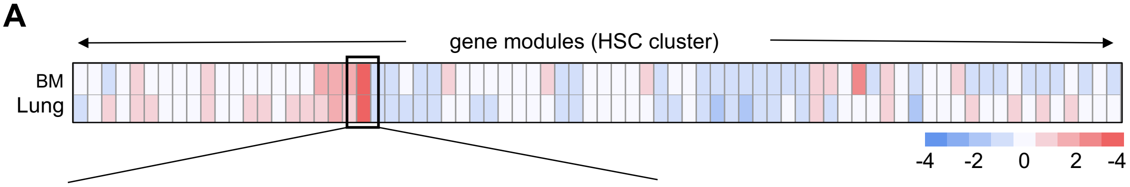

Module 54: *SPINK2, HOPX, TPT1, SELL, AREG, AVP, AJ009632.2, HLA-E, SOCS2, HEMGN, C1QTNF4, ADGRG6, BST2, CSF3R, TXNIP, MLLT3, RFLNB, GLIPR1, TCEAL2, CXCL2, FLT3, RCSD1, NAAA, BAALC, ATP8B4, TFEC, LPCAT2, INPP4B, LAIR1, ICAM3, ATP1B1, GUCY1A1, MMP7, PIK3IP1, TCTEX1D1, PDZK1IP1, PREX2, CALN1, PCDH9, TMEM163, BEX2, ELMO1, SLC2A5, BEX1, SORL1, GIMAP1, PTGER4, ABCB1, SMIM3, GSTM5, LYSMD2, MIR155HG, SULT1C4, RIC3, CDADC1, SSBP2, AGPS, LINC00891, CBX2, IL18, NAALADL1, PTAFR, STARD3, MSRB3, A1BG, CYSLTR1, SOCS2-AS1, NPM2, MBOAT7, SCN9A, CPLX1, GSTM2, ADAM28, CLDN15, AC004130.2, BCAS4, ITGA6, HLF, ACOT11, ROBO4, GIMAP5, TNFSF4, PRKCH, BEX5, GPAT3, SH3D21, F2RL1, PTP4A3, PGM5, KRT18, KRT8, KIAA1257, LPIN1, RHPN1, HOXA6, MMP28, COL24A1, TSPAN2, C9orf43, ALS2, PLAG1, AP000547.3, AC007336.1, PANX2, MYO5C, DENND6B, FAM47E, CYB561D2.1, WAKMAR2, LINC00893, OXT, CACNA1F, STX17-AS1, LY6G5C, PRX, BEND7, AL359220.1, UBOX5, COL9A2, GRAP, FCMR, TNFRSF10D*

B

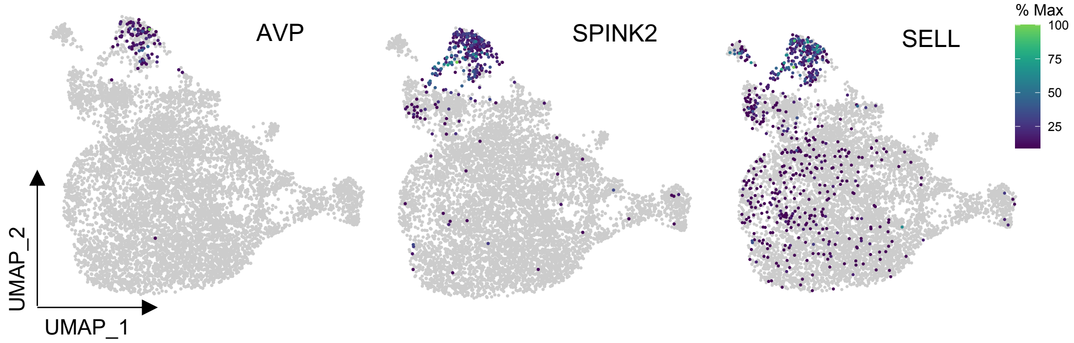

Supplemental Figure 7

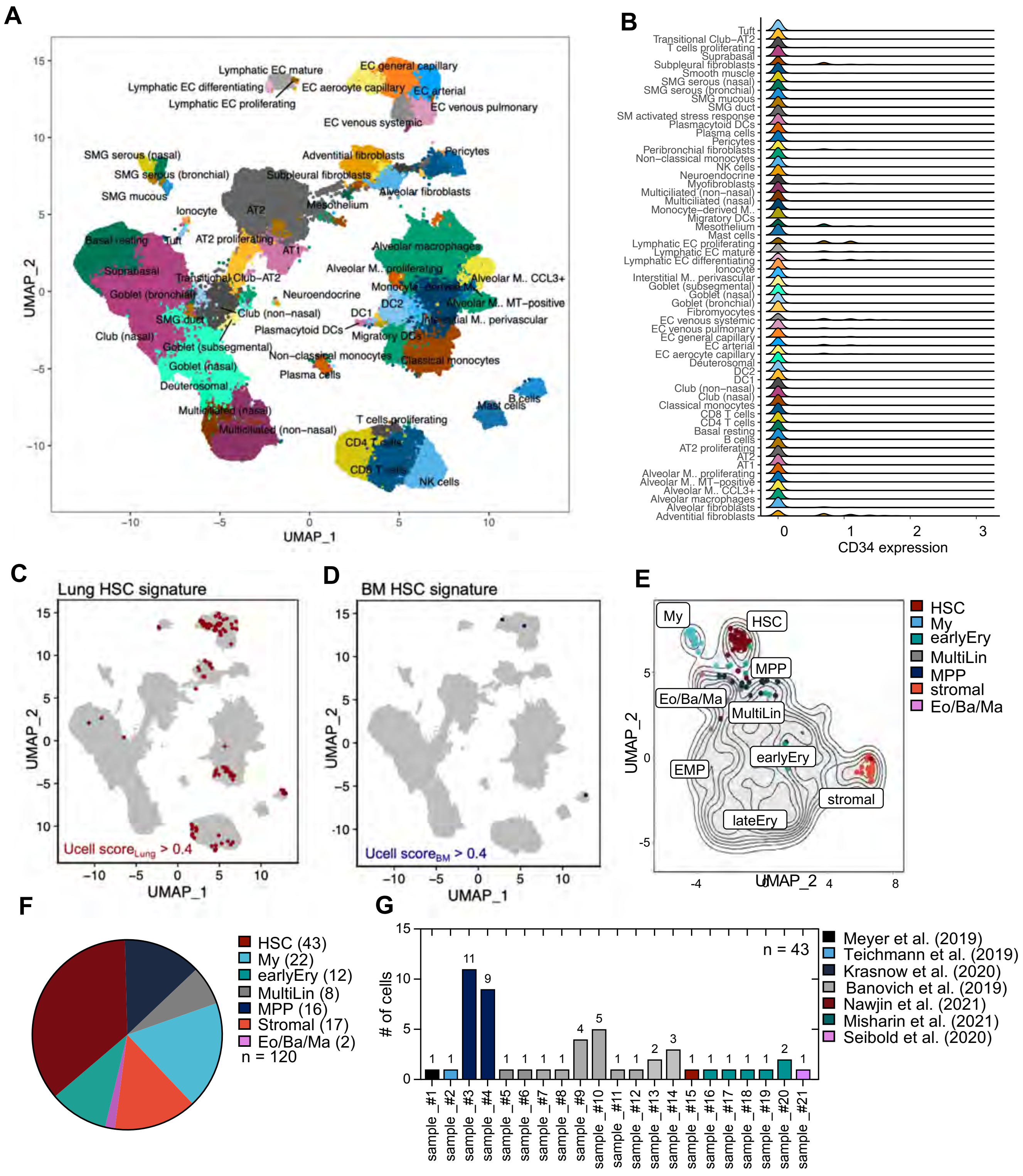

Supplemental Figure 8

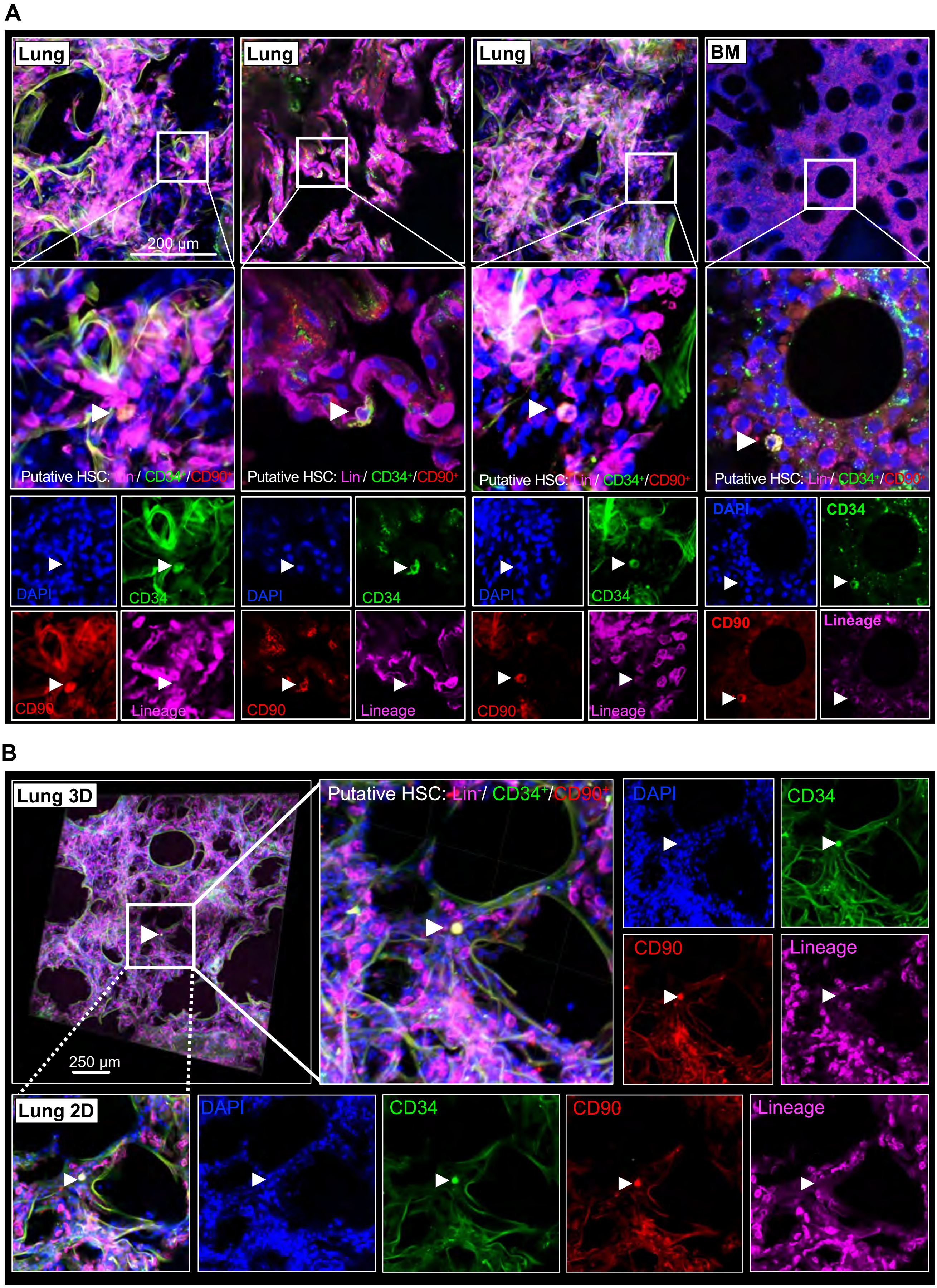

Supplemental Figure 9

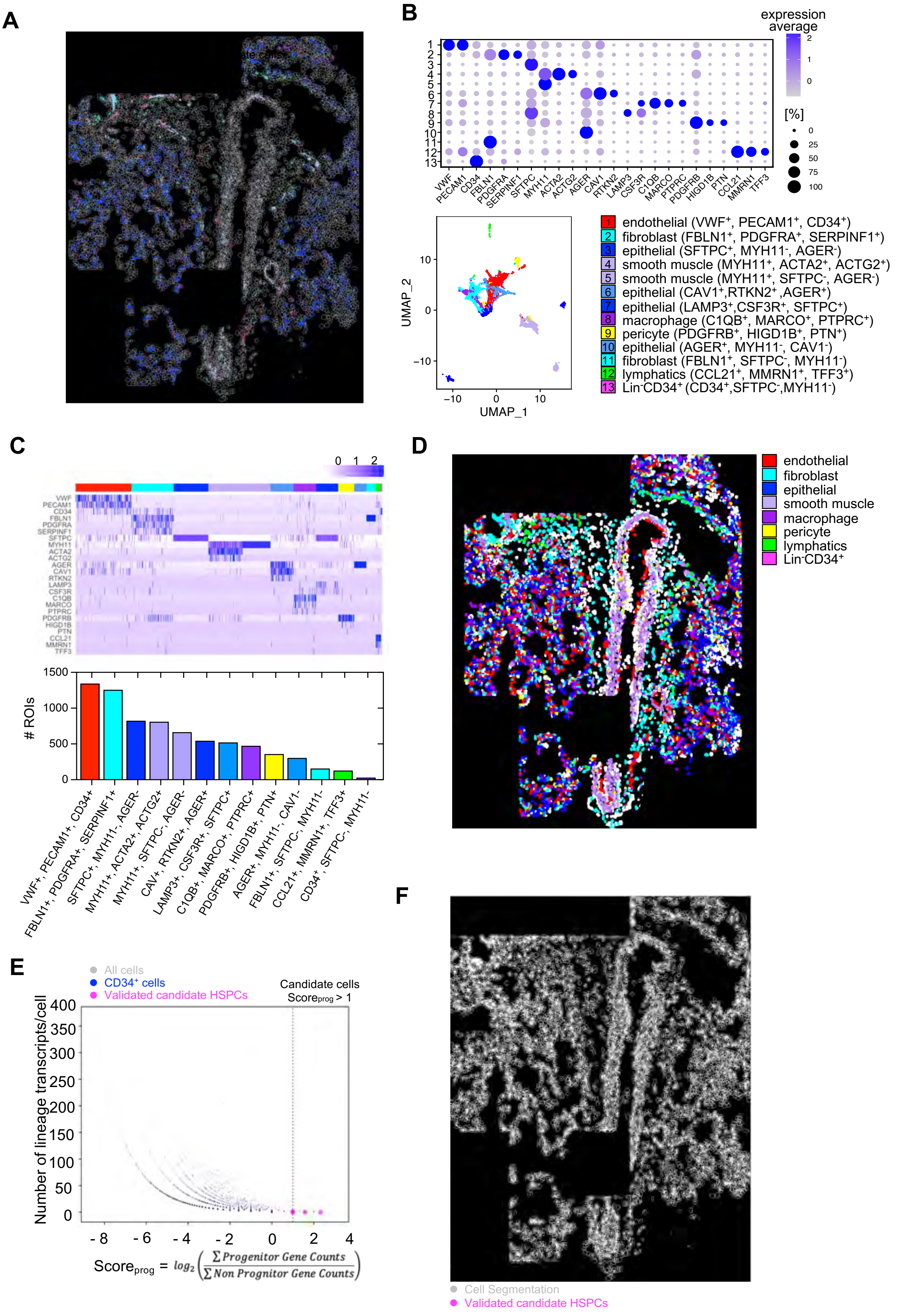

Supplemental Figure 10

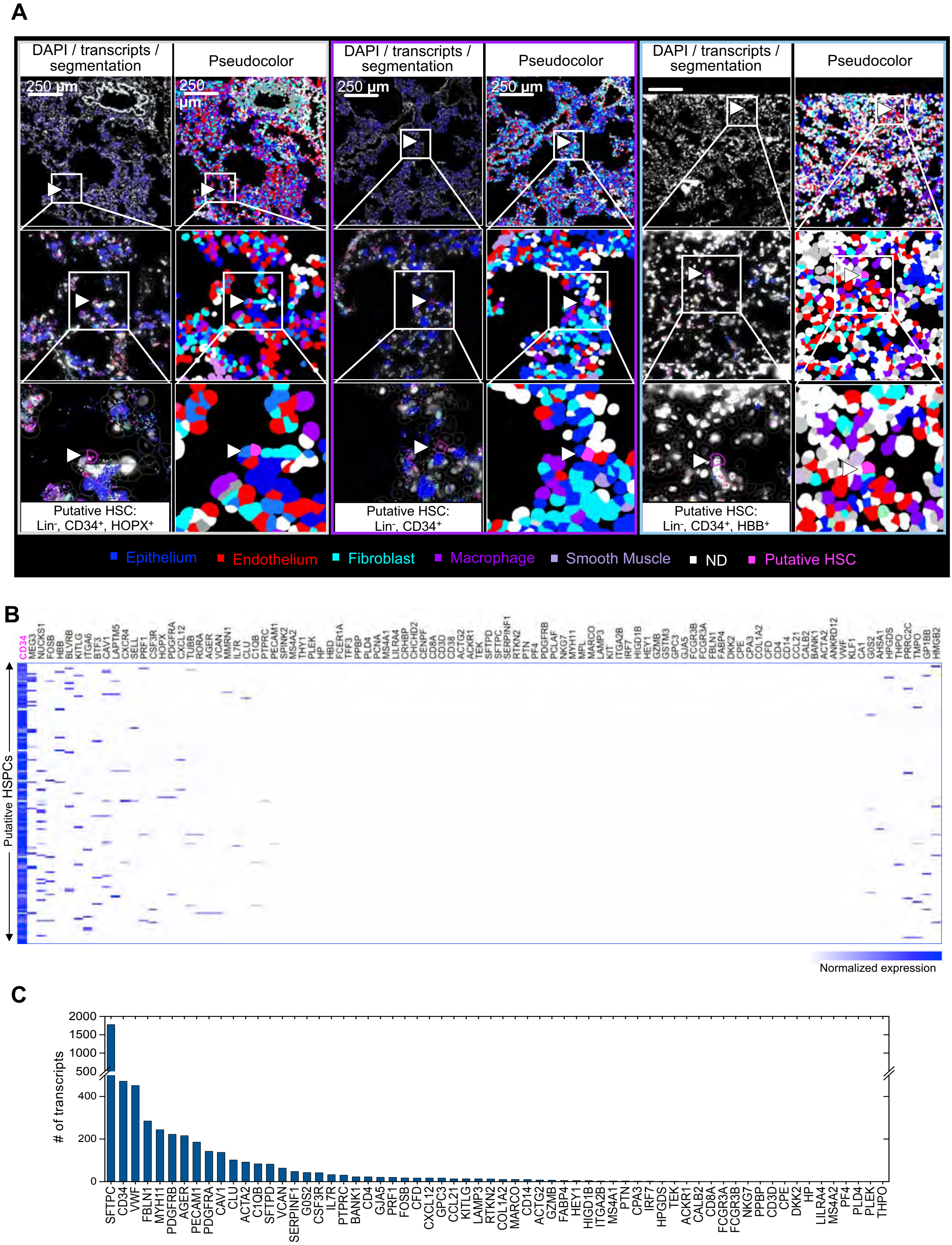

Supplemental Figure 11

Supplemental References

1. Nicolini FE, Cashman JD, Hogge DE, Humphries RK, Eaves CJ. NOD/SCID mice engineered to express human IL-3, GM-CSF and Steel factor constitutively mobilize engrafted human progenitors and compromise human stem cell regeneration. *Leukemia.* 2004;18(2):341-347.
2. Belluschi S, Calderbank EF, Ciaurro V, et al. Myelo-lymphoid lineage restriction occurs in the human haematopoietic stem cell compartment before lymphoid-primed multipotent progenitors. *Nat Commun.* 2018;9(1):4100.
3. Amend SR, Valkenburg KC, Pienta KJ. Murine Hind Limb Long Bone Dissection and Bone Marrow Isolation. *J Vis Exp.* 2016(110).
4. Kang HM, Subramaniam M, Targ S, et al. Multiplexed droplet single-cell RNA-sequencing using natural genetic variation. *Nat Biotechnol.* 2018;36(1):89-94.
5. Dobin A, Davis CA, Schlesinger F, et al. STAR: ultrafast universal RNA-seq aligner. *Bioinformatics.* 2013;29(1):15-21.
6. Hafemeister C, Satija R. Normalization and variance stabilization of single-cell RNA-seq data using regularized negative binomial regression. *Genome Biol.* 2019;20(1):296.
7. Hao Y, Hao S, Andersen-Nissen E, et al. Integrated analysis of multimodal single-cell data. *Cell.* 2021;184(13):3573-3587.e3529.
8. McGinnis CS, Murrow LM, Gartner ZJ. DoubletFinder: Doublet Detection in Single-Cell RNA Sequencing Data Using Artificial Nearest Neighbors. *Cell Syst.* 2019;8(4):329-337.e324.
9. Korsunsky I, Millard N, Fan J, et al. Fast, sensitive and accurate integration of single-cell data with Harmony. *Nat Methods.* 2019;16(12):1289-1296.
10. Van der Auwera GA, Carneiro MO, Hartl C, et al. From FastQ data to high confidence variant calls: the Genome Analysis Toolkit best practices pipeline. *Curr Protoc Bioinformatics.* 2013;43:11.10.11-11.10.33.
11. DePristo MA, Banks E, Poplin R, et al. A framework for variation discovery and genotyping using next-generation DNA sequencing data. *Nat Genet.* 2011;43(5):491-498.
12. Li H. A statistical framework for SNP calling, mutation discovery, association mapping and population genetical parameter estimation from sequencing data. *Bioinformatics.* 2011;27(21):2987-2993.
13. Hay SB, Ferchen K, Chetal K, Grimes HL, Salomonis N. The Human Cell Atlas bone marrow single-cell interactive web portal. *Exp Hematol.* 2018;68:51-61.
14. Travaglini KJ, Nabhan AN, Penland L, et al. A molecular cell atlas of the human lung from single-cell RNA sequencing. *Nature.* 2020;587(7835):619-625.
15. Cao J, Spielmann M, Qiu X, et al. The single-cell transcriptional landscape of mammalian organogenesis. *Nature.* 2019;566(7745):496-502.
16. McDavid A, Finak G, Chattopadyay PK, et al. Data exploration, quality control and testing in single-cell qPCR-based gene expression experiments. *Bioinformatics.* 2013;29(4):461-467.
17. Finak G, McDavid A, Yajima M, et al. MAST: a flexible statistical framework for assessing transcriptional changes and characterizing heterogeneity in single-cell RNA sequencing data. *Genome Biol.* 2015;16:278.
18. Borcherding N, Vishwakarma A, Voigt AP, et al. Mapping the immune environment in clear cell renal carcinoma by single-cell genomics. *Commun Biol.* 2021;4(1):122.
19. Subramanian A, Tamayo P, Mootha VK, et al. Gene set enrichment analysis: a knowledge-based approach for interpreting genome-wide expression profiles. *Proc Natl Acad Sci U S A.* 2005;102(43):15545-15550.

20. Bunis DG, Andrews J, Fragiadakis GK, Burt TD, Sirota M. dittoSeq: universal user-friendly single-cell and bulk RNA sequencing visualization toolkit. *Bioinformatics.* 2021;36(22-23):5535-5536.
21. Andreatta M, Carmona SJ. UCell: Robust and scalable single-cell gene signature scoring. *Comput Struct Biotechnol J.* 2021;19:3796-3798.
22. Frankish A, Diekhans M, Ferreira AM, et al. GENCODE reference annotation for the human and mouse genomes. *Nucleic Acids Res.* 2019;47(D1):D766-D773.
23. Yates AD, Achuthan P, Akanni W, et al. Ensembl 2020. *Nucleic Acids Res.* 2020;48(D1):D682-D688.
24. Marçais G, Kingsford C. A fast, lock-free approach for efficient parallel counting of occurrences of k-mers. *Bioinformatics.* 2011;27(6):764-770.
25. Gans JD, Wolinsky M. Improved assay-dependent searching of nucleic acid sequence databases. *Nucleic Acids Res.* 2008;36(12):e74.
26. Rodriguez JM, Rodriguez-Rivas J, Di Domenico T, Vázquez J, Valencia A, Tress ML. APPRIS 2017: principal isoforms for multiple gene sets. *Nucleic Acids Res.* 2018;46(D1):D213-D217.
27. Schmidt U, Weigert M, Broaddus C, Myers G. Cell Detection with Star-convex Polygons. Medical Image Computing and Computer Assisted Intervention (MICCAI 2018); 2018; Granada, Spain.
28. Bankhead P, Loughrey MB, Fernández JA, et al. QuPath: Open source software for digital pathology image analysis. *Sci Rep.* 2017;7(1):16878.
29. Tosti L, Hang Y, Debnath O, et al. Single-Nucleus and In Situ RNA-Sequencing Reveal Cell Topographies in the Human Pancreas. *Gastroenterology.* 2021;160(4):1330-1344.e1311.
30. Palla G, Spitzer H, Klein M, et al. Squidpy: a scalable framework for spatial omics analysis. *Nat Methods.* 2022;19(2):171-178.
31. Deprez M, Zaragosi LE, Truchi M, et al. A Single-Cell Atlas of the Human Healthy Airways. *Am J Respir Crit Care Med.* 2020;202(12):1636-1645.
32. Goldfarbmuren KC, Jackson ND, Sajuthi SP, et al. Dissecting the cellular specificity of smoking effects and reconstructing lineages in the human airway epithelium. *Nat Commun.* 2020;11(1):2485.
33. Habermann AC, Gutierrez AJ, Bui LT, et al. Single-cell RNA sequencing reveals profibrotic roles of distinct epithelial and mesenchymal lineages in pulmonary fibrosis. *Sci Adv.* 2020;6(28):eaba1972.
34. Madissoon E, Wilbrey-Clark A, Miragaia RJ, et al. scRNA-seq assessment of the human lung, spleen, and esophagus tissue stability after cold preservation. *Genome Biol.* 2019;21(1):1.
35. Misharin AV, Budinger GRS. Targeting the Myofibroblast in Pulmonary Fibrosis. *Am J Respir Crit Care Med.* 2018;198(7):834-835.
36. Morse C, Tabib T, Sembrat J, et al. Proliferating SPP1/MERTK-expressing macrophages in idiopathic pulmonary fibrosis. *Eur Respir J.* 2019;54(2).
37. Schupp JC, Adams TS, Cosme C, Jr., et al. Integrated Single-Cell Atlas of Endothelial Cells of the Human Lung. *Circulation.* 2021;144(4):286-302.
38. Vieira Braga FA, Kar G, Berg M, et al. A cellular census of human lungs identifies novel cell states in health and in asthma. *Nat Med.* 2019;25(7):1153-1163.
